# Presynaptic spinophilin tunes neurexin signalling to control active zone architecture and function

**DOI:** 10.1038/ncomms9362

**Published:** 2015-10-16

**Authors:** Karzan Muhammad, Suneel Reddy-Alla, Jan H Driller, Dietmar Schreiner, Ulises Rey, Mathias A. Böhme, Christina Hollmann, Niraja Ramesh, Harald Depner, Janine Lützkendorf, Tanja Matkovic, Torsten Götz, Dominique D. Bergeron, Jan Schmoranzer, Fabian Goettfert, Mathew Holt, Markus C. Wahl, Stefan W. Hell, Peter Scheiffele, Alexander M. Walter, Bernhard Loll, Stephan J. Sigrist

**Affiliations:** 1 Freie Universität Berlin, Institute for Biology/Genetics, Takustrasse 6, Berlin 14195, Germany; 2 NeuroCure, Charité, Charitéplatz 1, Berlin 10117, Germany; 3 Freie Universität Berlin, Institut für Chemie und Biochemie /Strukturbiochmie, Takustrasse 6, Berlin D-14195, Germany; 4 Biozentrum, University of Basel, Klingelbergstrasse 50-70, Basel 4056, Switzerland; 5 Leibniz Institut für Molekulare Pharmakologie, Robert-Roessle-Strasse 10, Berlin 13125, Germany; 6 Department of NanoBiophotonics, Max Planck Institute for Biophysical Chemistry, Am Fassberg 11, Göttingen 37077, Germany; 7 VIB Center for the Biology of Disease, Herestraat 49, Leuven 3000, Belgium

## Abstract

Assembly and maturation of synapses at the *Drosophila* neuromuscular junction (NMJ) depend on trans-synaptic neurexin/neuroligin signalling, which is promoted by the scaffolding protein Syd-1 binding to neurexin. Here we report that the scaffold protein spinophilin binds to the C-terminal portion of neurexin and is needed to limit neurexin/neuroligin signalling by acting antagonistic to Syd-1. Loss of presynaptic spinophilin results in the formation of excess, but atypically small active zones. Neuroligin-1/neurexin-1/Syd-1 levels are increased at *spinophilin* mutant NMJs, and removal of single copies of the *neurexin-1, Syd-1* or *neuroligin-1* genes suppresses the spinophilin-active zone phenotype. Evoked transmission is strongly reduced at *spinophilin* terminals, owing to a severely reduced release probability at individual active zones. We conclude that presynaptic spinophilin fine-tunes neurexin/neuroligin signalling to control active zone number and functionality, thereby optimizing them for action potential-induced exocytosis.

## Abstract (web summary)

Synaptic assembly depends on trans-synaptic Neurexin/Neuroligin signalling. Here, Muhammad *et al*. show that Spinophilin, a pre-synaptic scaffolding protein, interacts with Neurexin, in competition with Syd-1, to regulate the formation and function of synaptic active zones at *Drosophila* neuromuscular junctions.

## Graphical abstract

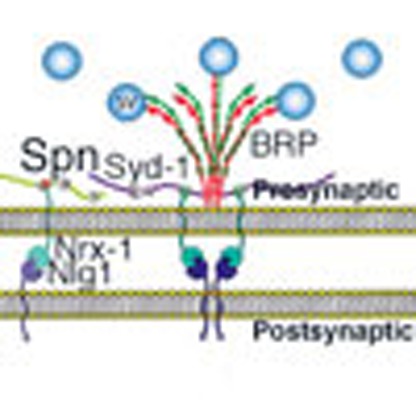

Chemical synapses release synaptic vesicles (SVs) at specialized presynaptic membranes, so-called active zones (AZs), which are characterized by electron-dense structures, reflecting the presence of extended molecular protein scaffolds. These AZ scaffolds confer stability and facilitate SV release^1^. Importantly, at individual AZs, scaffold size is found to scale with the propensity to engage in action potential-evoked release^2^,^3^,^4^. An evolutionarily conserved set of large multi-domain proteins operating as major building blocks for these scaffolds has been identified over the last years: Syd-2/Liprin-α, RIM, RIM-binding-protein (RBP) and ELKS family proteins (of which the the *Drosophila* homologue is called Bruchpilot (BRP))^1^,^5^,^6^,^7^. However, how presynaptic scaffold assembly and maturation are controlled and coupled spatiotemporally to the postsynaptic assembly of neurotransmitter receptors remains largely unknown, although trans-synaptic signalling via Neurexin-1 (Nrx-1)–Neuroligin-1 (Nlg1) adhesion molecules is a strong candidate for a conserved ‘master module' in this context, based on Nrx-Nlg signalling promoting synaptogenesis *in vitro*, synapses of rodents^8^,^9^, *Caenorhabditis elegans*^10^ and *Drosophila*^11^,^12^,^13^,^14^,^15^,^16^. With respect to scaffolding proteins, Syd-1 was found to promote synapse assembly in *C. elegans*^5^, *Drosophila*^17^ and rodents^18^. In *Drosophila*, the Syd-1-PDZ domain binds the Nrx-1 C terminus and couples pre- with postsynaptic maturation at nascent synapses of glutamatergic neuromuscular junctions (NMJs) in *Drosophila* larvae. Syd-1 cooperates with Nrx-1/Nlg1 to stabilize newly formed AZ scaffolds, allowing them to overcome a ‘threshold' for synapse formation^13^. Additional factors tuning scaffold assembly, however, remain to be identified. We show here that the conserved scaffold protein spinophilin (Spn) is able to fine-tune Nrx-1 function by binding the Nrx-1 C terminus with micromolar affinity via its PDZ domain. In the absence of presynaptic Spn, ‘excessive seeding' of new AZs occurred over the entire NMJ due to elevated Nrx-1/Nlg1 signalling. Apart from structural changes, we show that Spn plays an important role in neurotransmission since it is essential to establish proper SV release probability, resulting in a changed ratio of spontaneous versus evoked release at *Spn* NMJ terminals.

## Results

### Presynaptic Spn restricts the AZ number

**Figure 1:**
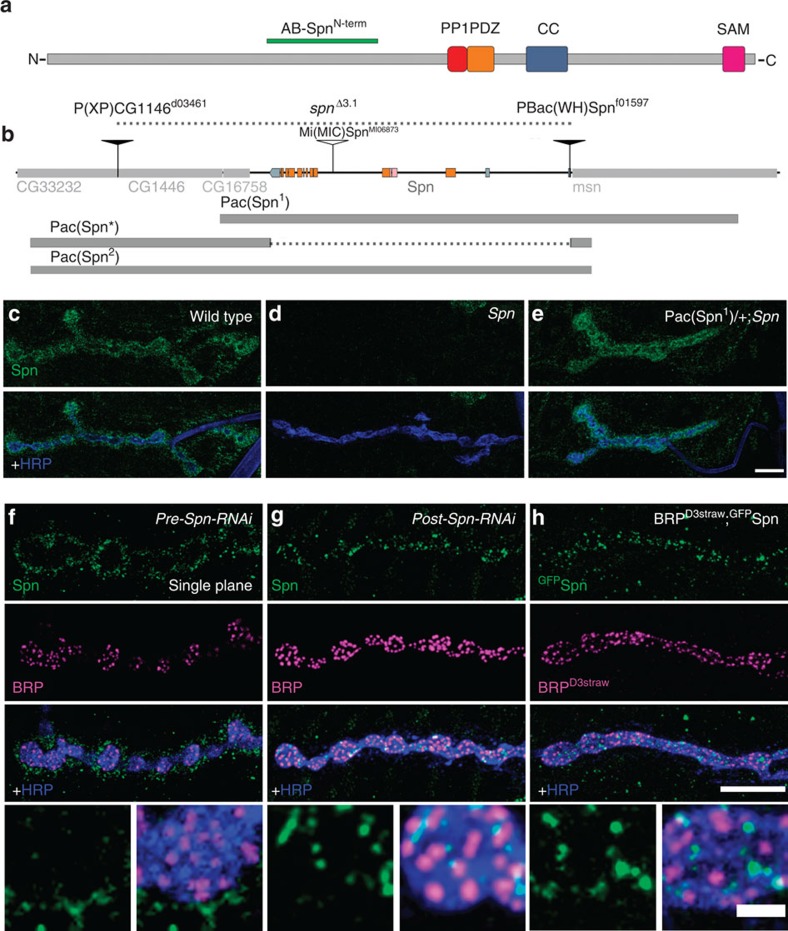
Characterization of the *Drosophila* *spn* locus. (**a**) Domain structure of Spn: protein phosphatase 1 (PP1) binding motif, PDZ domain, coiled coil (CC) domain and sterile alpha motif (SAM) domain. (**b**) Organization of the *spn* locus. Transposon lines used in the generation of *Spn* mutants, positions covered by the Pacman constructs indicated on a genomic map of *Spn*. (**c**) Immunostaining with Spn antibody (green) and HRP antibody (blue) at control NMJs, (**d**) at *Spn* null NMJs (**e**) and *Spn* null NMJs with a genomic rescue construct. (**f**) Presynaptic knockdown of Spn leaves the HRP boundaries devoid of Spn protein. (**g**) Postsynaptic knockdown of Spn using a muscle driver line reveals discrete clusters of Spn within the presynaptic terminals. (**h**) Presynaptic co-labelling of ^GFP^Spn together and BRP D3^strawberry^ using a motor neuron driver. Scale bars, 10 μm; 2 μm in magnified images.

Glutamatergic NMJs of *Drosophila* larvae continuously expand to meet the requirements of the growing muscle fibres by adding new release sites (or synapses) to their structure^19^,^20^. These synapses are characterized by a single presynaptic AZ opposed by a single postsynaptic density (PSD) composed of glutamate receptors (GluRs). AZ formation is initiated by both Syd-1 and Liprin-α clusters and finalized by the incorporation of BRP^21^. Here we used the *Drosophila* NMJ model system to search for factors restricting the number of BRP scaffolds. To this end, a set of proteins and their known binding partners, which we previously detected in immunoprecipitation experiments against BRP^22^, were suppressed by RNA interference (RNAi) restricted to presynaptic motor neurons. RNAi-induced presynaptic knockdown of the only *Drosophila* homologue of the Neurabin/Spn family caused an increase of AZ numbers at the NMJ (Fig. 1a; Supplementary Fig. 1a–e). Simultaneously, the total area of postsynaptic GluRs increased (Supplementary Fig. 1b–e).

Motivated by this result, we generated a *Spn* null allele using Flippase-mediated trans-deletion of FRT sites with two transposon lines flanking the *spn* locus, resulting in a complete deletion of the Spn-encoding sequence (*spn*^*Δ3.1*^) (Fig. 1b). Genomic PCR^23^ was used to validate the elimination of the entire *spn* locus. Animals died in pupal stages when we put the *spn*^Δ3.1^ chromosome in trans to a large deficiency (*spn*^*Δ3.1*^*/dfBSc116*, from hereafter *Spn*). Neurabin/Spn family proteins in rodents are strongly expressed in postsynaptic spines^24^,^25^ and are also found in presynaptic compartments^26^,^27^. Our presynaptic Spn knockdown clearly affected AZ scaffold formation, pointing towards a presynaptic role for Spn at *Drosophila* NMJs. To validate this hypothesis, and to determine Spn localization, we raised a polyclonal antibody against a fusion protein from the Spn N-terminal region (Anti-Spn^Nterm^, Fig. 1a; green bar). The Spn antibody robustly stained wild-type NMJs, but the signal was lost in *Spn* mutant larvae (Fig. 1c,d). Staining was restored after crossing in a genomic Spn rescue construct (Pac(Spn^1^)), proving the specificity of the NMJ Spn antibody signal (Fig. 1e). To characterize the localization of endogenous Spn in pre- versus postsynaptic compartments, we expressed the Spn-RNAi transgene in either the pre- or postsynaptic compartment of the NMJ using specific Gal4-driver lines. Motoneuron-driven presynaptic RNAi left the anti-Spn staining intact at the bouton periphery, but removed the staining within the *horseradish peroxidase* (HRP) signal, which outlines the neuronal membrane (Fig. 1f). Muscle-driven postsynaptic RNAi made the Spn staining surrounding the boutons vanish. However, the signal inside the presynaptic boutons (Fig. 1g) remained unchanged. When a ^GFP^Spn fusion construct was co-expressed with the AZ marker BRP-D3^Straw^ within the motoneurons^21^, presynaptic Spn formed discrete clusters, often found adjacent to BRP-labelled AZ scaffolds (Fig. 1g,h). This pattern was very similar to the residual endogenous Spn staining found remaining after the expression of RNAi in the postsynaptic muscle (Fig. 1g). Thus, Spn localizes to both pre- and postsynaptic compartments at larval NMJs. Presynaptic Spn localizes close to presynaptic AZ scaffolds.

**Figure 2:**
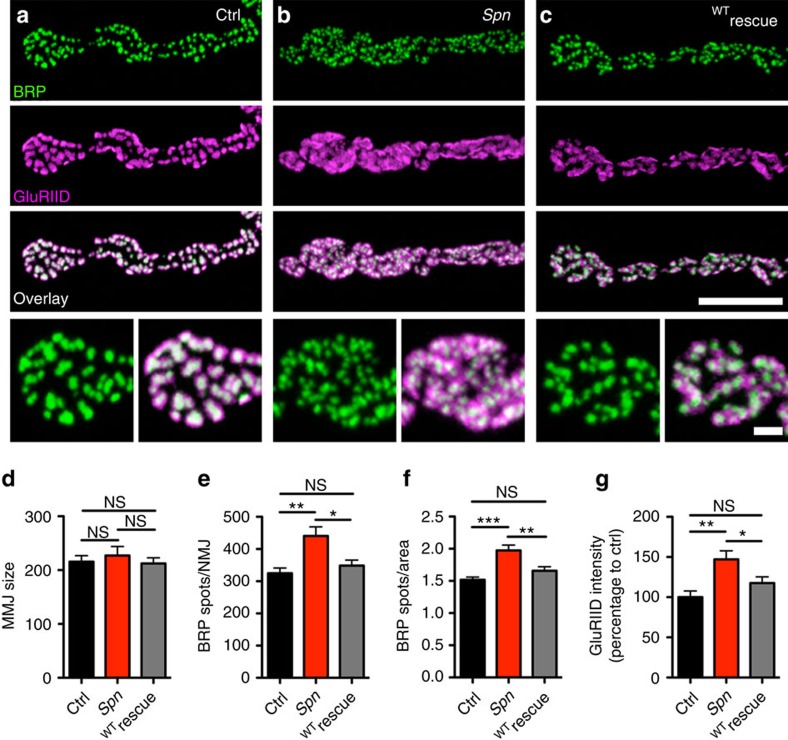
Presynaptic Spn limits NMJ AZ numbers. (**a**–**c**) Projected confocal stacks of NMJs (muscle 4), labelled against BRP (BRP^Nc82^, green) and GluRIID (magenta). (**d**) NMJ sizes measured using HRP labelling. (**e**) Numbers of AZ scaffolds per NMJ measured using BRP^Nc82^ labelling (Ctrl: 324.8±16.29, *n*=14; *Spn*: 440.5±28.4 *n*=13; neuronal ^WT^Spn cDNA expression (^WT^rescue): 348.4±17.45, *n*=14; Ctrl versus *Spn P<*0.01, (*U*=25); Ctrl versus ^WT^rescue: *P*>0.05, (*U*=79); *Spn* versus ^WT^rescue: *P<*0.05, (*U*=38)). (**f**) AZ scaffold densities (spots per μm^2^): ^WT^Spn cDNA expression (Ctrl: 1.5±0.04, *n*=14; *Spn*: 1.97±0.08, *n*=13; ^WT^rescue: 1.65±0.6, *n*=14; Ctrl versus *Spn P<*0.001, (*U*=13); Ctrl versus ^WT^Spn rescue: *P*>0.05; (*U*=57); *Spn* versus ^WT^rescue: *P<*0.01, (*U*=36)). (**g**) Integrated GluRIID intensity is higher in *Spn* (Ctrl: 100±7.6, *n*=14; *Spn*: 147.1±10.74, *n*=13; ^WT^rescue: 117.6±7.6, *n*=14; Ctrl versus *Spn*: *P<*0.01, (*U*=31); Ctrl versus ^WT^rescue: *P*>0.05, (*U*=65); *Spn* versus ^WT^rescue: *P<*0.05, (*U*=53). All tests are Mann–Whitney *U*-test, values are mean±s.e.m., NS, not significant; *P≤0.05; **P≤0.01; ***P≤0.001. Scale bar, 10 or 1.5 μm in magnified images.

Subsequently, we analysed the role of Spn in synaptic organization at developing NMJs, using the null allele (*Spn*) we created (Fig. 1b). Detailed analysis of *Spn* NMJs revealed that AZ scaffold densities increased. Postsynaptic GluR (GluRIID) labelling^28^ was also strikingly increased (Fig. 2a,b). We expressed two different but overlapping genomic pacman transgenes^29^ containing the full *spn* locus (Pac(Spn^1&2^); Fig. 1b) in the null allele mutant background to prove the specificity of the *Spn* null phenotype. Both genomic constructs fully rescued adult viability and, importantly, the NMJ phenotypes of *Spn*. In addition, deletion of a stretch encoding the Spn open reading frame within the genomic construct of Pac(Spn^2^), named Pac(Spn*), abolished rescue activity (data not shown). We further tested a semi-lethal transposon insertion within the *spn* locus (Mi(Mic)Spn^MI06873^), which we found to significantly reduce anti-Spn staining. The latter mutant showed NMJ phenotypes similar, but somewhat weaker, than those observed in *Spn* null larvae (Supplementary Fig. 2a–e). Taken together, we show that loss of Spn affects the synaptic structure of the NMJ. We quantified relevant structural parameters using BRP/GluRIID/HRP co-stainings to further characterize this phenotype (Fig. 2d–g). Average NMJ size (visualized via HRP) was not significantly changed in the *Spn* null background. Similarly, but more pronounced than in the RNAi experiments, the densities of presynaptic AZs (BRP cluster numbers normalized to synaptic HRP area) were significantly increased in *Spn* when compared with controls (Fig. 2d,e). We re-expressed the protein using a neuronal driver line *elav(x)-C155-gal4* in the *Spn* null background to test whether this was due to a loss of presynaptic Spn. Indeed, presynaptic expression of Spn complementary DNA (cDNA) effectively re-established normal AZ densities (Fig. 2c–e). By contrast, postsynaptic (that is, muscle) expression of Spn in the null background appeared to have no effect (data not shown). Moreover, the postsynaptic phenotype of increased GluR fields was reverted towards normal levels on presynaptic Spn expression (Fig. 2f). Thus, presynaptic Spn restricts both the dimensions of the PSD, as well as the number of juxtaposed presynaptic BRP scaffolds. The BRP scaffold is tightly associated with Ca^2+^ channels and RBP, another structural component of the AZ scaffold^30^. Numbers of Ca^2+^ channel clusters and RBP clusters were also increased at *Spn* terminals (Supplementary Fig. 3a–f). By contrast, cysteine string protein, a SV protein, appeared unchanged when compared with controls (Supplementary Fig. 3d–h). Taken together, these data show that *Spn* terminals have a specific increase in the number of AZ scaffolds.

### AZ scaffolds lacking Spn remain small

**Figure 3:**
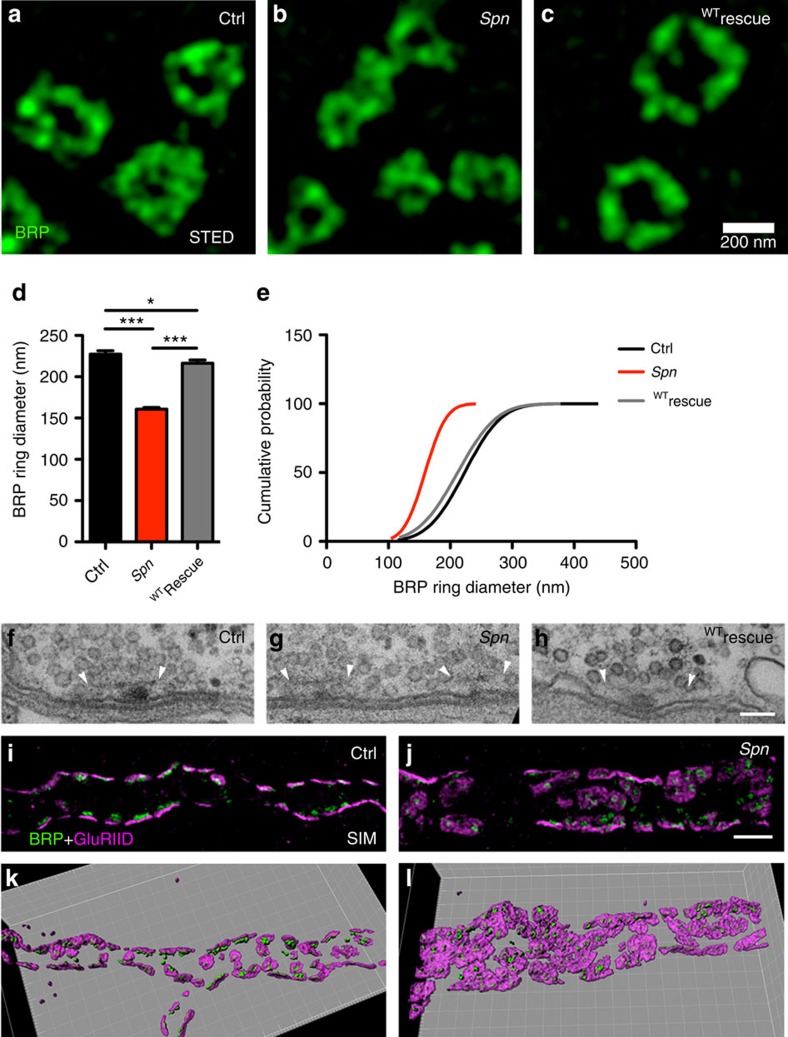
**Ultrastructural analyses of** ***Spn*** **NMJ synapses.** (**a**–**c**) STED-derived BRP rings are atypically small in *Spn* terminals. (**d**,**e**) Quantification of BRP ring diameters. Control: 227.5±4 nm, *n*=168; *Spn*: 160.8±2 nm, *n*=178; ^WT^rescue: 216.3±3.9 nm, *n*=156; Kruskal–Wallis test with Dunn's multiple comparison test (*K*=186). *P≤0.05; **P≤0.01; ***P≤0.001. Error bars: mean±s.e.m. (**f**,**h**) Electron microscopy of presynaptic electron-dense projections (T-bars) of (**f**) control boutons, (**g**) *Spn* boutons with more, but smaller T-bars; the *Spn* phenotype which can be rescued by presynaptic re-expression of Spn (**h**). Arrowheads indicate the edges of T-bars platforms. (**i**,**j**) Structured Illumination (SIM) analysis of WT and *Spn* NMJs. Co-labelling of GluRIID and BRP^Nc82^ for wild-type (**i**) and *Spn* (**j**) NMJs show excessive accumulations of GluRs at *Spn* NMJs with arrays of small BRP scaffolds converging on enlarged GluR fields. (**k**,**l**) 3D rendering of SIM images shown above. Scale bars: STED, 200 nm; EM, 100 nm; SIM, 2 μm.

Confocal images suggested that individual presynaptic AZ scaffolds, as identified by their BRP spots, were atypically small at *Spn* terminals. However, confocal resolution (∼250 nm) is not sufficient to reliably quantify AZ scaffold size. Thus, we turned to stimulated emission depletion (STED) microscopy operating with ≈45 nm lateral resolution^21^,^31^ to visualize AZ scaffolds in their planar orientation (Fig. 3a–c). Analysing the longest peak-to-peak axes through individual AZs revealed that the diameters of BRP AZ scaffolds were substantially reduced in *Spn* mutants, while presynaptic Spn re-expression restored normal AZ size (Fig. 3a–e).

In summary, a larger number of smaller presynaptic AZ scaffolds are forming in the absence of presynaptic Spn. Electron microscopic (EM) analysis consistently revealed smaller but otherwise normal T-bars (Fig. 3f,h, arrowheads; Supplementary Fig. 4a–e). In some cases, two of these small T-bars converged (juxtaposed) into one common large postsynaptic compartment, identified by a region in which pre- and postsynaptic membranes were tightly apposed (Fig. 3g).

GluRs at wild-type NMJ synapses localize at postsynaptic membranes opposed to presynaptic AZs. As mentioned above (Fig. 2), individual GluR clusters were atypically enlarged in *Spn*. As details of the GluR organization may not be resolved by standard confocal imaging, we used three-dimensional structured illumination microscopy (3D SIM) with an isotropic resolution of ≈120 nm^32^. This provides a significant improvement in optical resolution along the *z*-axis, while STED only increases the *x–y* resolution. Therefore, SIM allowed us to resolve the 3D organization of GluR fields relative to the AZs. Consistent with the EM analysis, *Spn* NMJs showed extended, often continuous receptor fields, juxtaposed to several small AZs, with a clear increase in the area of the postsynaptic compartment labelled with GluRs (Fig. 3i–l).

### Increased Nrx-1 signalling mediates the *Spn* phenotype

**Figure 4:**
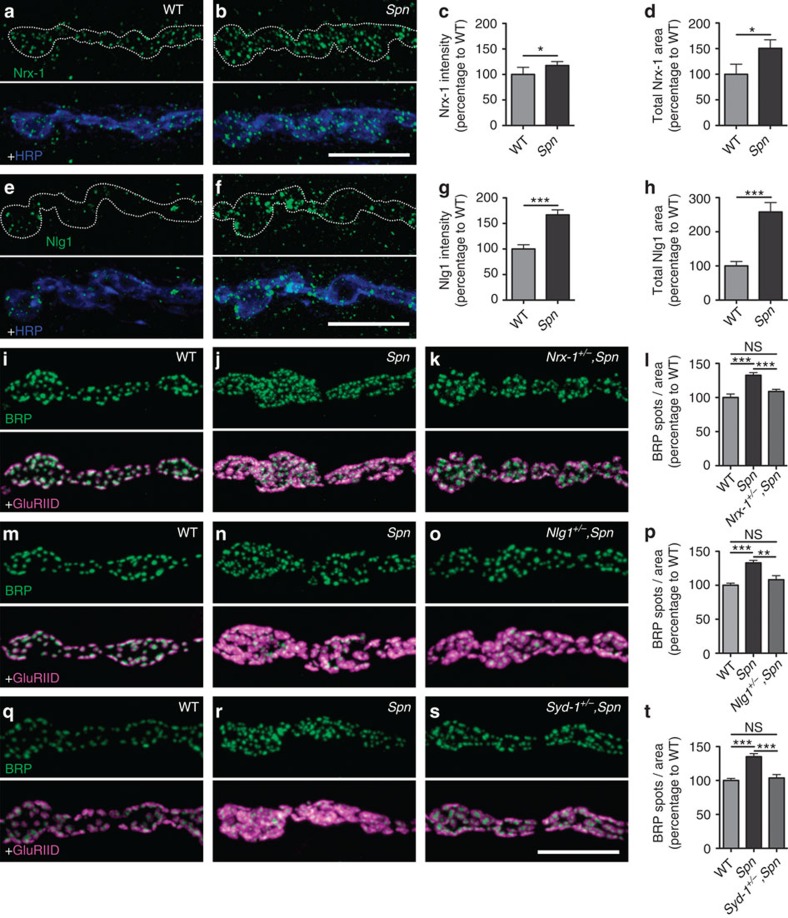
Spn regulates trans-synaptic signalling. All mutant tests Mann–Whitney *U*-test, values are mean±s.e.m., NS, not significant; *P≤0.05; **P≤0.01; ***P≤0.001. (**a**–**d**) Upregulation of Nrx-1 levels at *Spn* mutant NMJs. (**a**,**b**) Muscle 4 NMJs of wild-type and *Spn* larvae immunostained for Nrx-1. (**c**,**d**) Quantification of Nrx-1 signals. Total Nrx-1 covered area (a.u.): wild type 100±19.63, *n*=19; *Spn* 150.7±16.6, *n*=19; wild type versus *Spn P<*0.05, (*U*=92); Nrx-1 intensity (a.u.): wild type 100±14.12, *n*=19; *Spn*: 117.5±7.6, *n*=19; wild type versus *Spn*, *P<*0.05, *U*=108. (**e**–**h**) Upregulation of Nlg1 levels at *Spn* NMJs. (**e**,**f**) Wild type and *Spn* larvae immunostained for Nlg1. (**g**,**h**) Quantifications of Nlg1 signals. Total Nlg1 covered area (a.u.). Wild type 100±13.21, *n*=19; *Spn* 257±27, *n*=19; wild type versus *Spn P<*0.001, (*U*=42). Nlg1 intensities (a.u.): wild type 100±8.3, *n*=19; *Spn*: 166.7±9.8, *n*=19; wild type versus *Spn*, *P<*0.001, (*U*=33). (**i**–**t**) Genetic interaction analysis of *Spn* phenotypes. (**i**–**l**) Genetic suppression of *Spn* phenotypes by Nrx-1 (**i**–**k**). NMJs immunostained for BRP^Nc82^ and GluRIID. (**l**) Quantification of BRP spot densities at NMJs. Wild type 100±5, *n*=11; *Spn* 132.7±3.8, *n*=12; *Nrx-1*^*241*^/+*, Spn*: 108.9±3.1; wild type versus *Spn*, *P<*0.001, (*U*=10); wild type versus *Nrx-1*^*241*^*/+, Spn*, *P*>0.05, (*U*=50); *Spn* versus *Nrx-1*^*241*^*/+, Spn*, *P<*0.001, (*U*=17). (**m**–**p**) Genetic suppression of *Spn* phenotype by Nlg1; (**p**) Quantification of BRP spot densities. Wild type 100±3.1, *n*=10; *Spn* 132.9±3.7, *n*=11; *Nlg1*^*2.3*^*/+, Spn* 108.1±6; wild type versus *Spn*, *P<*0.001, (*U*=3); wild type versus *Nlg1*^*2.3*^*/+, Spn*, *P*>0.05, (*U*=37); *Spn* versus *Nlg1*^*2.3*^*/+, Spn*, *P<*0.01, (*U*=20). (**q**–**t**) Genetic suppression by Syd-1. (**t**) wild type: 100±3, *n*=10; *Spn*: 135.1±4.6, *n*=13; *Syd-1*^*3.4*^*/+, Spn*: 103.7±5; wild type versus *Spn*, *P<*0.001; (*U*=4). Wild type versus *Syd-1*^*3.4*^*/+, Spn P*>0.05; (*U*=57). *Spn* versus *Syd-1*^*3.4*^*/+, Spn*, *P<*0.001; (*U*=21). Scale bars, 10 μm.

PSDs of *Drosophila* NMJs contain two subtypes of GluR complexes, distinguished by the incorporation of either receptor subunit GluRIIA or GluRIIB^28^. Immature wild-type PSDs contain more GluRIIA than IIB, while GluRIIB incorporation occurs during subsequent PSD maturation, revealed by *in vivo* imaging^33^. We recently discovered that *Nlg1*, *Nrx-1* and *Syd-1* mutants share a specific deficit in the incorporation of GluRIIA receptors into the PSD driving ‘early' PSD growth^13^. In contrast, here we observed a threefold increase of GluRIIA intensity at *Spn* terminals, probably responsible for the overgrowth of the postsynaptic GluR fields, while GluRIIB levels remained unchanged (Supplementary Fig. 5a–e). Thus, lack of Spn apparently results in an opposite phenotype to Nrx-1 signalling pathway mutants (*Nrx-1*, *Nlg1, Syd-1*), which show fewer but larger and often mis-shapen AZ scaffolds^13^,^15^,^16^. To further investigate a possible antagonistic relationship between Spn and Nrx-1/Nlg1, we investigated whether Nrx-1 levels were changed at *Spn* terminals, using an antibody detecting endogenous Nrx-1 (ref. 15). We observed a significant increase in the levels of Nrx-1 (measured either as the total integrated fluorescence from the anti-Nrx-1 label, or total area of Nrx-1 clusters normalized to synaptic HRP area; Fig. 4a–d). We next asked whether this increase in Nrx-1 could promote Nrx-1 signalling. To test this, we first evaluated the levels of Nlg1 and Syd-1 in *Spn* mutants. We found that the level of both proteins increased at *Spn* NMJs (Fig. 4e–h; Supplementary Fig. 6a–g). However, Fasciclin-II (another cell adhesion molecule unrelated to the Nrx-1/Nlg1 signalling pathway^34^) was unchanged (Supplementary Fig. 7a,b). Next, to confirm that Nrx-1 signalling is directly responsible for the generation of more AZs at *Spn* terminals, a single copy of the nrx-1 gene (allele *Nrx-1*^*241*^; ref. 15) was removed from the *Spn* background. This manipulation in wild type background had no detectable effect on NMJ and AZ organization (ref. 15; data not shown). Strikingly, AZ numbers were reduced to wild type levels after removing a single *nrx-1* gene copy from the *Spn* background (Fig. 4i–l). The AZ assembly and maturation mediated by Nrx-1 depends on both muscle expressed (postsynaptic) Nlg1 (refs 11, 35) and presynaptic Syd-1. In fact, removing a single *nlg1* gene copy in *Spn* null background (*nlg*^*2.3*^; ref. 11) suppressed the *Spn* phenotype (Fig. 4m–p). Furthermore, removing a single gene copy of *syd-1* also suppressed the *Spn* phenotype (Fig. 4q–t). We went on to analyse the functional relationship between Spn and Syd-1; both are presynaptically expressed scaffold proteins containing a PDZ domain.

### Antagonism of Spn and Syd-1 for Nrx-mediated synapse assembly

We previously found that Nrx-1 levels are decreased in *Syd-1* mutants, but stabilized on re-expression of Syd-1. Moreover, previous fluorescence recovery after photobleaching (FRAP) analysis showed elevated mobility of Nrx-1^GFP^ in a *Syd-1* mutant background^13^. As Nrx-1 and Syd-1 clusters in *Spn* were upregulated (Fig. 4a–d;Supplementary Fig. 6a–g), we asked whether it was possible that the motility of Nrx-1 was altered in *Spn* mutants by performing FRAP experiments on Nrx-1^GFP^. We found a delayed recovery and, thus, reduced motility of Nrx-1 in the *Spn* null background (Supplementary Fig. 6i–l). At the same time, lack of *Drosophila* CASK (Caki), another scaffolding protein that binds to the Nrx-1 C terminus^36^,^37^, did not show any noticeable effect on Nrx-1 motility (Supplementary Fig. 6). Moreover, the recovery of Syd-1^GFP^ clusters appeared to be unchanged at *Spn* terminals (even though the cluster density was increased) (Supplementary Fig. 6i–k). Thus, Spn-mediated Nrx-1 motility is apparently not connected to altered Syd-1 motility.

We further investigated whether, as suggested by the Nrx-1 FRAP data, Syd-1 and Spn would operate in a competitive manner. Consequently, we revisited our previous finding that overexpression of Syd-1 within motoneurons results in co-expressed Nrx-1^GFP^ being recruited into AZs^13^. However, when Spn was also co-overexpressed with Nrx-1^GFP^ and ^mStrawberry^Syd-1, both the Nrx-1^GFP^ level and ^mStrawberry^Syd-1 level at AZs dropped (Nrx1^GFP^ intensity in wild-type background: 1.0±0.06, *n*=20; Nrx1^GFP^ intensity in the presence of overexpressed Spn: 0.8±0.04, *n*=19; *P<*0.01; Mann–Whitney *t*-test (*U*=113). ^mStraw^Syd-1 intensity in wild-type background: 1.0±0.04, *n*=20; ^mStraw^Syd-1 intensity in the presence of Spn: 0.76±0.05; *P<*0.01; Mann–Whitney *t*-test (*U*=75)). Thus, Spn gain-of-function might influence Nrx-1, antagonistic to the Spn loss-of-function phenotype (Fig. 4b; Supplementary Fig. 6a–g). In fact, AZ sizes on Spn overexpression were slightly (but significantly) increased over controls (Ctrl: 222±3, *n*=108; ^GFP^Spn: 246±4.5, *n*=160; ctrl versus ^*GFP*^*Spn OE P<*0.001; Student's *t*-test).

### The Spn-PDZ domain interacts with Nrx-1 C terminus

**Figure 5:**
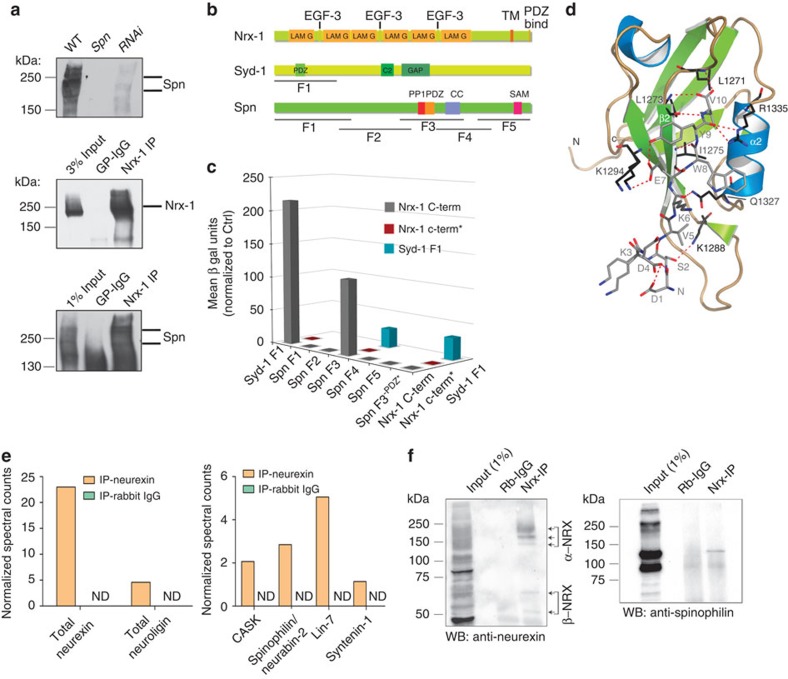
Spn interacts directly with the Nrx-1 C-term. (**a**) Western blot analysis of larval head extracts from wild type, *Spn* and pan-neuronal *elav(x)-c155-gal4* driven Spn-RNAi show the specificity of our custom anti-Spn^Nterm^ antibody. Immunoblot of Nrx-1 immunoprecipitate (IP) from a *Drosophila* head fractionation enriched for AZ proteins (Methods). Spn can be detected using a Nrx-1 Co-immunoprecipitation (co-IP), but is absent when a control immunoglobulin G is used (IgG). (**b**) Domain structures of Nrx-1, Syd-1 and Spn. Nrx-1 possesses extracellular laminin G (LAM G) and epidermal growth factor (EGF)-3 domains, as well as a PDZ binding motif at the C terminus. Syd-1 comprises an N-terminally located PDZ domain, a C2 domain and a putative RhoGAP domain. Spn domain structure and the boundaries of fragments used in Y2H experiments (F1-F5) (Supplementary Fig. 7). (**c**) Quantitative liquid Y2H assay for binding of individual Spn fragments (and Syd-1 F1) with the Nrx-1 C terminus. Fragment 3, containing the PDZ domain, binds strongly to the Nrx1 C-term. Binding is fully abolished when a point mutation is introduced into the ligand-binding site of the Spn-PDZ domain, or when the last five amino acid residues of the Nrx-1 C-term (Nrx-1 c-term*) are deleted. (**d**) A structural representation of the Spn-PDZ interacting with the Nrx-1 C-term peptide. The C-terminal Nrx peptide is shown in grey using a stick representation. Residues on Spn-PDZ that interact with the Nrx peptide are highlighted in black. Red dashed lines indicate potential hydrogen, bonds with a distance cut-off of ≤3.3 Å. (**e**) Mass-spectometric analysis of protein complexes immunoprecipitated from mouse whole brain homogenate using Nrx-1 antibody. (**f**) Western blot analysis showing the Nrx antibody effectively co-IPs Spn (see Supplementary Fig. 12).

We performed immunoprecipitation experiments from *Drosophila* head extracts^22^, using antibodies against Nrx-1 (refs 13, 15), to test whether Spn and Nrx-1 might be part of a common complex. Western blot analysis with the anti-Spn antibody specifically detected bands in the range of ∼200 kD, validating the specificity of our custom-made anti-Spn antibodies (Fig. 5a; upper panel). Using Nrx-1 antibodies, which robustly immunoprecipitated Nrx-1 (Fig. 5a; middle panel), Spn could be co-immunoprecipitated, but was absent in negative controls which used an irrelevant IgG (Fig. 5a; lower panel). We performed a yeast two-hybrid (Y2H) analysis using a C-terminal fragment of Nrx-1 to screen against different fragments of Spn to investigate a direct Nrx-1/Spn interaction (Fig. 5b,c). As a control, we included a Syd-1 fragment, which we had previously shown to interact with Nrx-1 (ref. 13). Semiquantitative Y2H analysis uncovered a strong and specific interaction between the cytosolic part of Nrx-1 (hereafter termed Nrx-1 C-term) and a 500 amino acid region of Spn containing the PDZ domain (Spn-F3) (Spn-F3 × Nrx-1 C-term in Fig. 5c). The fact that the overlapping constructs F2 and F4 (Fig. 5b) did not show any interaction narrowed down the possible interacting stretch to a region comprising only the PP1 and the PDZ domains. These domains are present in all Spn family members and are highly conserved between fly, worm and rodent (Supplementary Fig. 8a). The Nrx-1 C-term/Spn-F3 interaction was eliminated after deleting the last five amino acids of the Nrx-1 C terminus. In addition, introduction of a point mutation^38^ in the Spn-PDZ domain (in the ligand-binding pocket) which abolishes ligand binding, also abolished the interaction (Fig. 5c). Thus, the very C-terminal PDZ-binding motif of Nrx-1 interacts directly with PDZ domains found in both Spn and Syd-1. To characterize the binding of Nrx-1 C-term to the Spn-PDZ domain at atomic resolution, we turned to X-ray crystallography. We solved the structure of PDZ domain containing residues 1,258–1,347 of Spn in complex with the last 10 C-terminal residues of Nrx-1 (at 1.2 A° resolution) (Fig. 5d; Supplementary Fig. 8; Supplementary Table 1). The Spn-PDZ domain shares the characteristic canonical fold of PDZ domains, which is composed of six β-strands and two α-helices^39^. According to its specificity for C-terminal peptides, Spn-PDZ is a class II PDZ domain, recognizing the signature motif X–Ψ–X–Ψ (X, unspecified; and Ψ, hydrophobic amino acid residue). We found the peptide-binding groove to be flanked by a β-strand (β2) and an α-helix (α2). The Nrx-1 peptide binds in an anti-parallel mode, with main chain/main chain hydrogen bonding to β2 of the Spn-PDZ. The carboxylate of the Nrx-1 peptide is hydrogen bonded to backbone amides of L1271 and L1273 in Spn-PDZ (Fig. 5d;Supplementary Table 2). Further interactions are established with the side chains of Spn-PDZ residues residing on β4 and α2 (Fig. 5d). In addition, we observed an inter-peptide interaction that might be important for stabilizing the peptide conformation. We investigated the binding thermodynamics of the Nrx-1 C-term peptide to the PDZ domains of Spn or Syd-1 using isothermal calorimetry (ITC). The Syd-1-PDZ domain showed higher affinity binding (Kd 5 μM) than the Spn-PDZ domain (50 μM) (Supplementary Fig. 8e,f). Both Spn-PDZ domains and Nrx-1 C-termini are highly conserved between *Drosophila* and rodents (Supplementary Fig. 8a,c). In fact, an *in vitro* pull-down experiment effectively precipitated both the *Drosophila* Spn-PDZ and rat Spn-PDZ using the respective Nrx-1 peptides (Supplementary Fig. 9a). To validate an *in vivo* interaction between Spn and Nrxs in rodents, we performed co-immunoprecipitation experiments from mouse whole brain lysates using a newly generated affinity-purified pan-Nrx antibody (Supplementary Fig. 9b). We analysed the co-imunoprecipitated proteins by mass spectrometry. Nlg, Spn and several additional synaptic PDZ-domain-containing proteins known to interact with Nrxs could be detected in the Nrx immunoprecipitates, but not in precipitations with control IgGs (Fig. 5e). The presence of Spn/Nrx complexes was further confirmed by western blotting of the precipitates (Fig. 5f). Thus, we find that Spn/Nrx interactions show evolutionary conservation fully consistent with their shared sequence conservation.

### PDZ domain ligand binding of Spn controls AZ structure and function

**Figure 6:**
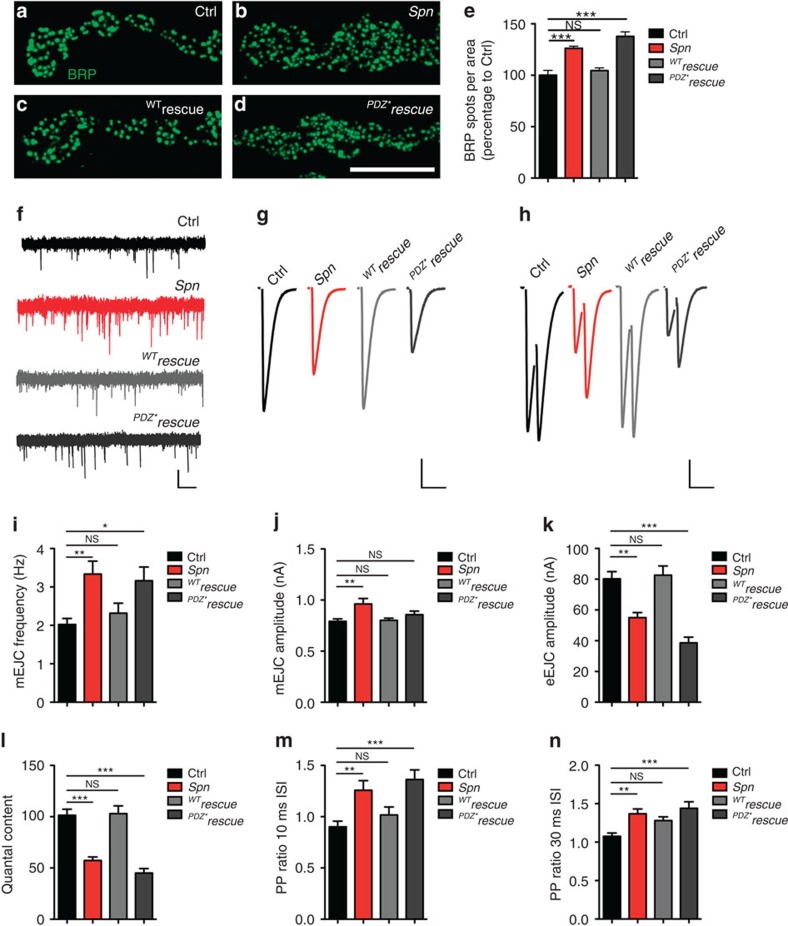
**Electrophysiological characterization of** ***Spn*** **NMJs.** (**a**–**d**) BRP^Nc82^ labelling in indicated genotypes. (**e**) Quantification of BRP spot densities in **a**–**d**, Ctrl: 100±4.6, *n*=6; *Spn*: 126.1±2.08, *n*=8; ^WT^rescue: 104.5±2.6, *n*=8; ^PDZ*^rescue: 137.8±4.45, *n*=7. Ctrl versus *Spn P<*0.001, (*U*=2). *Spn* versus ^WT^rescue *P<*0.001, (*U*=0.0). ^WT^rescue versus ^PDZ*^rescue P<0.001, (*U*=0). (**f**) Representative mEJCs traces. (**g**) Representative eEJCs traces. (**h**) Paired-pulse measurements with inter stimulus interval (ISI) of 10 ms; (**i**) Quantification of mEJC frequencies (Ctrl: 2.02±0.16, *n*=28; *Spn*: 3.33±0.34, *n*=15, *P<*0.01; ^WT^*rescue*: 2.32±0.26, *n*=16, *P*>0.05; ^PDZ*^rescue: 3.16±0.36, *n*=16, *P<*0.05. (**j**) Quantification of mEJC amplitudes (Ctrl: −0.78±0.03 nA, *n*=28; *Spn*: −0.96±0.05 nA, *n*=15, *P<*0.01; ^WT^rescue: −0.80±0.02 nA, *n*=16, *P*>0.05; ^PDZ*^rescue: −0.86±0.03 nA, *n*=15, *P*>0.05). (**k**) Quantification of eEJC amplitudes (Ctrl: −80.23±4.66 nA, *n*=28; *Spn*: −55.00±3.29 nA, *n*=24, *P<*0.01; ^WT^rescue: −82.58±6.0 nA, *n*=18, *P*>0.05; ^PDZ*^rescue: −38.66±3.67, *n*=18, *P<*0.01). (**l**) Quantification of quantal content (Ctrl: 101.4±5.89, *n*=28; *Spn*: 57.20±3.42, *n*=24, *P<*0.001; ^WT^rescue: 103.0±7.53, *n*=18, *P*>0.05; ^PDZ*^rescue: 45.13±4.29, *n*=18, *P<*0.001). (**m**) Quantification of the pair pulse ratio with an ISI of 10 ms. (Ctrl: 0.90±0.05, *n*=28; *Spn*: 1.26±0.09, *n*=22; *P<*0.01; ^WT^rescue: 1.01±0.08, *n*=18, *P*>0.05; ^PDZ*^rescue: 1.36±0.09, *n*=18, *P<*0.001). (**n**) Quantification of the paired-pulse ratio with a 30 ms ISI (Ctrl: 1.08 ±0.04, *n*=28; Spn: 1.37±0.06, *n*=21, *P<*0.01; ^WT^rescue: 1.28±0.05, *n*=18, *P*>0.05; ^PDZ*^rescue: 1.44±0.08, *n*=17, *P<*0.001). Statistics: one-way analysis of variance with Tukey's multiple comparison post test. All panels show mean±s.e.m., NS, not significant; *P≤0.05; **P≤0.01; ***P≤0.001. Scale bars: **a**–**d**, 10 μm; **f**, 1 nA/1 s; **g**,**h**, 20 nA/20 ms.

If binding of the Spn-PDZ domain to Nrx-1 was, in fact, functionally relevant, introducing the point mutation^13^,^38^ that interferes with Nrx-1 binding *in vitro* should compromise Spn function *in vivo*. Indeed, expression of the Spn cDNA containing the relevant point mutation (^PDZ*^Spn) no longer rescued the structural presynaptic AZ phenotype of *Spn* mutants. As expected, expression of wild-type cDNA (^WT^Spn; Fig. 2c) rescued the phenotype (Fig. 6a–e). Thus, interfering with ligand binding to the Spn-PDZ domain renders the protein incapable of limiting AZ numbers.

Finally, we investigated the physiological consequences of presynaptic Spn loss. We performed two-electrode voltage-clamp recordings (TEVC) to assay SV release. We observed a clear increase in the frequency of spontaneous SV release from *Spn* terminals, which dropped to normal rates when normal (^WT^Spn) was re-expressed in the presynaptic motoneuron (Fig. 6f,i). However, on expression of ^PDZ*^Spn under identical conditions, the frequencies of spontaneous release events remained high (Fig. 6f,i). The amplitudes of single spontaneous release events were significantly larger at *Spn* terminals (Fig. 6j), potentially reflecting the larger postsynaptic GluRIIA receptor fields described above (Fig. 2b; Supplementary Fig. 5; Fig. 3i–l). In contrast, release evoked by single action potentials was clearly decreased at *Spn* NMJs (Fig. 6g,k). Loss of Spn also altered synaptic short-term plasticity, in response to stimulation with a pair of action potentials (at 10- or 30-ms intervals). Here *Spn* NMJs displayed abnormal facilitation (Fig. 6h,m,n). Both defects were rescued by the presynaptic expression of ^WT^Spn, while expression of the PDZ*Spn again did not rescue. Altogether, these results suggest that Spn is not only responsible for the functional distribution of presynaptic AZ scaffolds but also plays an important role in SV release, and that the reduced evoked responses were not due to decreased postsynaptic sensitivity. In addition, a higher number of presynaptic AZs, as observed in *Spn* terminals, is in line with an increased number of spontaneous release events detected. However, the fact that evoked release is lowered is unexpected, raising the question of whether the additional AZs observed in *Spn* are sub-optimal for evoked release, but can maintain spontaneous release. To answer this question, we went on to investigate the function of Spn at the single AZ level.

### Spinophilin optimizes evoked release at single synapses

**Figure 7:**
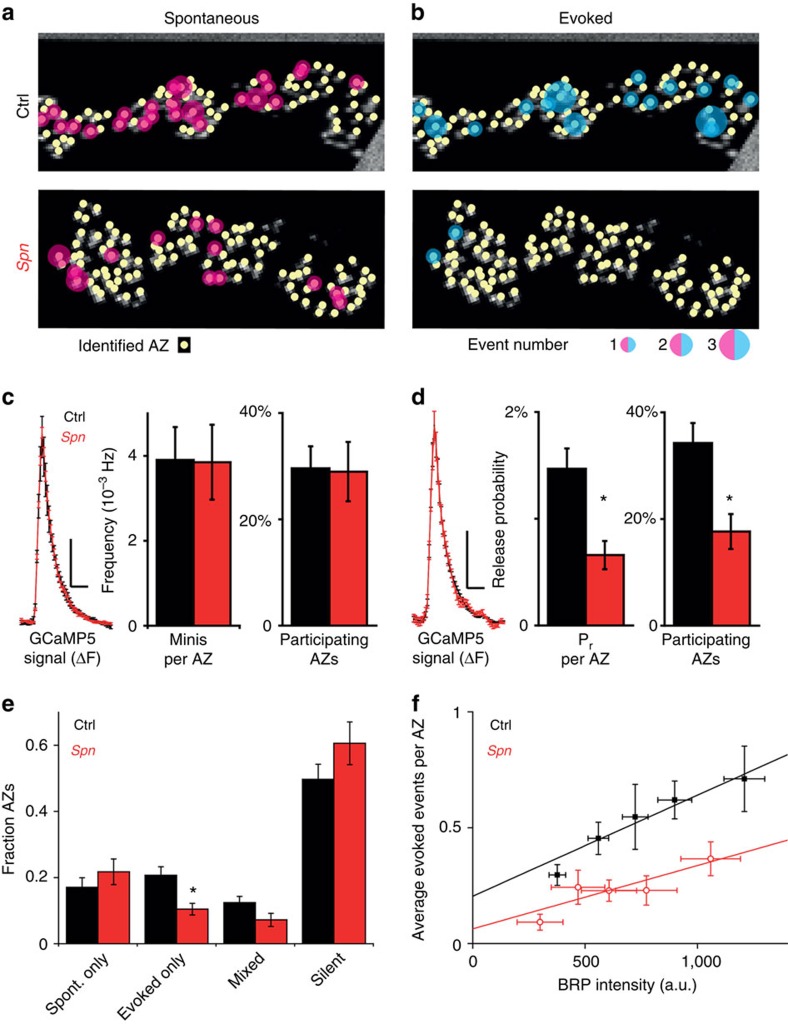
**Individual** ***Spn*** **AZs show normal spontaneous release, but lower probabilities for action potential-induced (evoked) release.** (**a**,**b**) Synaptic activity at control and *Spn* NMJs was first imaged for 100 s without stimulation. Subsequently, exocytosis was stimulated by 35 action potentials at 0.2 Hz. Images are montages of NMJ confocal scans showing staining for BRP^Nc82^. Spontaneous and evoked activities indicated by magenta and cyan circles, sizes reflect the number of events per AZ. (**c**) Local average postsynaptic GCaMP5 signals at *Spn* and control AZs in response to spontaneous release events (left traces). Frequencies of spontaneous (‘mini') events per AZ in *Spn* and controls very similar (centre bar graph), as is the fraction of AZs participating at least once in spontaneous activity (right bar graph). (**d**) Evoked release causes similar postsynaptic GCaMP5 signals at individual *Spn* and control AZs (left traces). The probability that an AZ shows release in response to a single action potential (pr) significantly reduced in *Spn* compared with controls (centre bar graph). The fraction of AZs responding at least once to stimulation also significantly reduced in *Spn* mutants AZs (right bar graph). (**e**) Categorization of AZs based on their activity pattern: (1) AZs exclusively active during spontaneous release (spont. only), (2) AZs exclusively responsive to AP-stimulation (evoked only), (3) AZs releasing both modes at least once (mixed) or (4) AZs not responding (silent). The fraction of 'evoked only‘ AZs was significantly reduced at *Spn* NMJs. (**f**) Reduced pr at *Spn* AZs is not secondary to lower BRP levels. AZs were binned with regard to their local BRP intensity and the average number of evoked events was plotted against the average BRP intensities (Supplementary Information File). Evoked events per AZ were correlated to local BRP levels in controls (black data points: experimental data, black line: linear fit, reduced *r*^2^=0.92) and, to a lesser extent, at *Spn* AZs (red data points: experimental data, red line: linear fit, reduced *r*^2^=0.69). Loss of Spn reduced evoked release more than expected by a mere reduction of BRP and both dependencies were best fit by different lines (F-test, *P*<0.05). Values are mean±s.e.m. Vertical/horizontal scale bars in **c**,**d**: 100 a.u./200 ms. Number of animals (*n*): Control: *n*=5, *Spn*: *n*=4. *P<0.05 in Mann–Whitney *U*-test.

The TEVC recordings sample release events over the whole NMJ of the respective muscle, but do not allow for the analysis of individual AZs. To investigate the latter, we used a recently developed assay employing post-synaptically expressed GCaMP to characterize the spatial and temporal dynamics of exocytotic events^2^,^3^,^40^. We imaged GCaMP responses to spontaneous exocytosis for 100 s (see Supplementary Movies 1 and 2 for examples) and, subsequently GCaMP response to action potential stimulation (35 action potentials given at 0.2 Hz, see Supplementary Movies 3 and 4 for examples). After recordings, larvae were fixed, stained against BRP and visualized using confocal microscopy. Alignment of these confocal images to the live movies (Supplementary Fig. 10; see methods for further details) allowed us to map activity at individual AZs (Fig. 7). Strikingly, spontaneous activity per AZ was not changed at *Spn* NMJs, suggesting that the net increase of spontaneous events observed in TEVC experiments is, indeed, due to an increase in synapse number rather than in their individual release rates (Fig. 7a,c). By contrast, the probability of evoked exocytosis was drastically reduced (Fig. 7b,d). However, the individual evoked GCaMP signals were indistinguishable between *Spn* and control NMJs (Fig. 7d). Consistent with our TEVC results, we found that loss of Spn changed the partitioning of AZs between these two discrete release modes: the fraction of AZs dedicated to evoked release was significantly reduced in *Spn* (Fig. 7e). Therefore, we conclude that even though Spn-deficient synapses participate in both modes of SV release, Spn is essential for establishing correct synaptic release probability, in agreement with the altered short-term plasticity we observed in our TEVC experiments (Fig. 6k–n). It was found recently that release probability at individual AZs correlated with the local levels of BRP^2^,^40^ which, as mentioned above, is reduced at *Spn* synapses (Fig. 3). Is the decrease in release probability at *Spn* synapses due to a reduction in their BRP levels? To address this question, we investigated the relationship between synaptic BRP and the number of release events evoked at single AZs^2^. We found that release probability was indeed positively correlated with BRP levels (Fig. 7f). Furthermore, the average number of release events evoked at *Spn* synapses also (but somewhat weaker) correlated with BRP level. However, as this relationship differed from that observed in control animals we can rule out the possibility that the effect is mediated solely through BRP reduction. Thus, we conclude that Spn is not only important for controlling synapse number and size, but also for optimizing action-potential-induced exocytosis by enhancing release probability at individual AZs.

## Discussion

The trans-synaptic dialogue between Nrx-1 and Nlg1 aids in the initial assembly, specification and maturation of synapses, and is a key component in the modification of neuronal networks^12^,^41^,^42^. Regulatory factors and processes that fine-tune and coordinate Nrx-1/Nlg1 signalling during synapse assembly process are currently under investigation. Our data indicate that *Drosophila* Spn-like protein acts presynaptically to attenuate Nrx-1/Nlg1 signalling and protects from excessive seeding of new AZ scaffolds at the NMJ. In *Spn* mutants, excessive AZs suffered from insufficient evoked release, which may be partly explained by their reduced size, and partly by a genuine functional role of Spn (potentially mediated via Nrx-1 binding).

**Figure 8:**
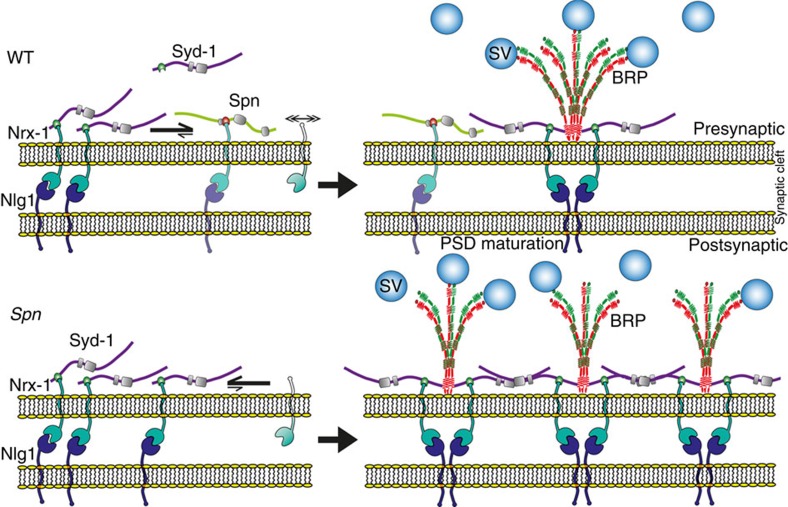
**Model describing the role of Spn in controlling the synaptogenic activity of Nrx-1 at** ***Drosophila*** **NMJs.** Spn acts antagonistically to Syd-1. In wild-type animals (upper panel), Nrx-1 interacts with postsynaptic Nlg1, as well as with either Syd-1 or Spn via PDZ domain-mediated interactions. In this way, trans-synaptic contact with Nlg1 can also steer postsynaptic assembly. The presence of Spn reduces the amount of Nrx-1 available for Syd-1 binding and, consequently, controls the number of AZs, by keeping the availability of critical proteins (BRP) below an assembly threshold. In addition, Nlg1-mediated postsynaptic assembly is also affected (not shown). In the absence of Spn (lower panel), Nrx-1 is less mobile and more efficiently recruited into complexes by Syd-1, resulting in the formation of excessive presynaptic AZ scaffolds.

In mice, loss of Spn (Neurabin II), one of the two Neurabin protein families present in mammals, was reported to provoke a developmental increase in synapse numbers^43^. While Spinophilin was found to be expressed both pre- and post-synaptically^26^,^27^, its function, so far, has only been analysed in the context of postsynaptic spines^43^,^44^,^45^,^46^. Given the conserved Spn/Nrx-1 interaction we report (Fig. 5), Spn family proteins might execute a generic function in controlling Nrx-1/Nlg1-dependent signalling during synapse assembly. We consistently find that Spn counteracts another multi-domain synaptic regulator, Syd-1, in the control of Nrx-1/Nlg1 signalling. Previous genetic work in *C. elegans* identified roles of Syd-1 epistatic to Syd-2/Liprin-α in synaptogenesis^5^,^47^. Syd-1 also operates epistatic to Syd-2/Liprin-α at *Drosophila* NMJs^17^,^48^. Syd-1 immobilizes Nrx-1 (ref. 13), positioning Nlg1 at juxtaposed postsynaptic sites, where it is needed for efficient incorporation of GluR complexes. Intravital imaging suggested an early checkpoint for synapse assembly, involving Syd-1, Nrx-1/Nlg1 signalling and oligomerization of Liprin-α in the formation of an early nucleation lattice^49^,^50^, which is followed later by ELKS/BRP-dependent scaffolding events^21^,^51^ (our model in Fig. 8, upper panel). As Spn promotes the diffusional motility of Nrx-1 over the terminal surface and limits Nrx-1/Nlg1 signalling, and as its phenotype is reversed by loss of a single gene copy of *nrx-1*, *nlg1* or *syd-1*, Spn displays all the features of a ‘negative' element mounting, which effectively sets the threshold for AZ assembly. As suggested by our FRAP experiments (Supplementary Fig. 6), Spn might withdraw a population of Nrx-1 from the early assembly process, establishing an assembly threshold that ensures a ‘typical' AZ design and associated postsynaptic compartments (Fig. 8). As a negative regulatory element, Spn might allow tuning of presynaptic AZ scaffold size and function (see below).

The *C. elegans* Spn homologue NAB-1 (*NeurABin1)* was previously shown to bind Syd-1 in cell culture recruitment assays^52^. We found consistent evidence for Syd-1/Nrx-1/Spn tripartite complexes in salivary gland experiments (Supplementary Fig. 11). Moreover, the PDZ domain containing regions of Spn and Syd-1 interacted in Y2H experiments (Fig. 5c). It would be interesting to dissect whether the interaction of Spn/Syd-1 plays a role in controlling the access of Nrx-1 to one or both factors. For *C. elegans* HSN synapses, a previous study^52^ showed that loss of NAB-1 results in a deficit of synaptic markers, such as Syd-1 and Syd-2/Liprin-α, while NAB-1 binding to F-actin was also found to be important for synapse assembly. Though at first glance rather contradictory to the results we describe in this study, differences might result from Chia *et al*.^52^ studying synapse assembly executed over a short time window, when partner cells meet for the first time^52^. In contrast, we used a model (*Drosophila* larval NMJs) where an already functional neuronal terminal adds novel AZs^17^,^21^. Despite our efforts, we were unable to demonstrate a role of F-actin in the assembly of AZs of late larval *Drosophila* NMJs. F-actin patches might be particularly important to establish the first synaptic contacts between partner cells. Both the study by Chia *et al*. and this study, however, point clearly towards important regulatory roles of Spn family members in the presynaptic control of synapse assembly.

Further, we describe a novel interaction between the Spn-PDZ domain and the intracellular C-term of Nrx-1 at the atomic level. Interestingly, we found that all functions of Spn reported in this study, structural as well as functional, were strictly dependent on the ligand-binding integrity of this PDZ domain. It is noteworthy that the Spn-PDZ domain binds other ligands as well, for example, Kalirin-7 and p70^S6K^ (refs 53, 54, 55), and further elucidation of its role as a signal ‘integrator' in synapse plasticity should be interesting. The fact that Nrx-1 levels were increased at *Spn* NMJs and, most importantly, that genetic removal of a single *nrx-1 gene* copy effectively suppressed the *Spn* AZ phenotype, indicates an important role of the Spn/Nrx-1 interaction in this context. Affinity of Spn-PDZ for the Nrx-1 C-term was somewhat lower than that of the Syd-1-PDZ, both in ITC and Y2H experiments (Fig. 5c). Nonetheless, overexpression of Spn was successful in reducing the targeting effect of Syd-1 on overexpressed Nrx-1^GFP^ (see above). It will be interesting to see whether this interaction can be differentially regulated, for example, by (de)phosphorylation.

It is worth noting that apart from Syd-1 and Spn, several other proteins containing PDZ domains, including CASK, Mint1/X11, CIPP and Syntenin^13^,^36^,^56^,^57^,^58^,^59^, were found to bind to the Nrxs C-termini (also see Fig. 5e,f). CASK was previously shown to interact genetically with Nrx-1, controlling endocytic function at *Drosophila* NMJs^36^. However, when we tested for an influence of CASK on Nrx-1^GFP^ motility using FRAP, genetic ablation of CASK had no effect (Supplementary Fig. 6). Thus, CASK function seemingly resembles neither Syd-1 nor Spn. Clearly, future work will have to address and integrate the role of other synaptic regulators converging on the Nrx-1 C-term. In particular, CASK (which displays a kinase function that phosphorylates certain motifs within the Nrx-1 C-term) might alternately control Spn- and Syd-1-dependent functions^37^. Presynaptic Nrx-1, through binding to postsynaptic Nlg1 at developing *Drosophila* NMJ terminals, is important for the proper assembly of new synaptic sites^11^,^13^,^15^,^36^. It is of note, however, that while mammalian Nrxs display robust synaptogenetic activity in cellular *in vitro* systems, direct genetic evidence for synaptogenetic activity of Nrxs in the mammalian CNS remained rather scarce. Triple knockout mice lacking all α-Nrxs display no gross synaptic defects at the ultrastructural level^60^,^61^. Future analysis will have to investigate whether differences here might be explained by specific compensation mechanisms in mammals; for example, by β-Nrxs, or other parallel trans-synaptic communication modules. Genuine functional deficits in neurotransmitter release were also observed after the elimination of presynaptic Spn. Elimination of ligand binding to the PDZ domain rendered the protein completely nonfunctional, without affecting its synaptic targeting. Thus, the *Spn* functional defects are likely to be mediated via a lack of Nrx-1 binding. Notably, ample evidence connects *Nrx-1* function with both the functional and structural maturation of *Drosophila* presynaptic AZs^8^,^16^,^41^,^62^,^63^. Our work now promotes the possibility that binding of Spn to Nrx-1 is important for establishing correct release probability, independent of absolute AZ scaffold size (Fig. 7). It is noteworthy that Nrx-1 function was previously shown to be important for proper Ca^2+^ channel function and, as a result, properly evoked SV release^60^. Thus, it will be interesting to investigate whether the specific functional contributions of Spn are mediated via deficits in the AZ organization of voltage-gated Ca^2+^ channels or Ca^2+^ sensors, such as synaptotagmin^64^,^65^,^66^. Taken together, we found an unexpected function for Spn in addition of AZs at *Drosophila* glutamatergic terminals, through the integration of signals from both the pre- and postsynaptic compartment. Given that we find the Spn/Nrx-1 interaction to be conserved from *Drosophila* to rodents, addressing similar roles of presynaptic Spn in mammalian brain physiology and pathophysiology might be informative.

## Methods

### Genetics and molecular cloning

Fly strains were reared under standard laboratory conditions^67^. Both male and female larvae were used for analysis in all experiments (except electrophysiological recordings, see below). The structure of the *spn*^*Δ3.1*^ allele eliminating the complete Spn locus, CG16758 (and partially deleting the CG45186 loci) was validated by genomic PCR^23^. The combination of *spn*^*Δ3.1*^ *in trans* with the deficiency chromosome dfBSc116 (Spn deficiency: Df) resulted in animals deficient in the Spn locus. Lethality in *Spn* was completely rescued by returning one copy of the genomic region of Spn in this mutant background. It is of note that another mutant allele of *Spn* was reported previously and was shown to be ‘semi-lethal'^68^; however, no functional analysis was performed in this study. *w^1118^* served as a genetic background for all experiments. Recombinations were verified using PCR or complementation analysis. The following recombination lines were used: for Syd-1(*dsyd-1^ex3.4^/+, spn^Δ3.1^/SpnDf*), Nrx-1 (*Nrx-1^241^/+, spn^Δ3.1^/SpnDf*) and Nlg1(*Nlg1^ex2.3^/+, spn^Δ3.1^/SpnDf*). Flies carrying UAS–green fluorescent protein (GFP)-tagged Nrx-1 (ref. 15), UAS–GFP or mStraw-tagged Syd-1 were described previously^13^. UAS-untagged or GFP-tagged Spn were obtained by recombining pUAST-attb-rfa and pUAST-attb–GFP–rfa with pENTR-Spn FL, respectively. The full-length Spn cDNA was cloned into pENTR from BDGP clone LD45234, via Spe1 and Kpn1 restriction sites, using primers 5′- ATGGATAGCGAAAAGGTGGCCAAAC -3′ and 5′- CTTCTTTTTGGCCGCCTTCTTCTC -3′.

A rabbit polyclonal anibody was raised against a 6 × His-tagged fusion protein of Spn N-term region (Fig. 1a, green bar). The corresponding expression construct was cloned after PCR with 5′- CACCAGCGTTCTCATCCAGTC -3′ and 5′- TTACACAATGTCCACGGCTTCA -3′ primers, and TOPO cloned into pENTR D-TOPO.

The point-mutated PDZ domain of Spn cDNA (^PDZ*^Spn cDNA) was constructed by circular PCR using primers: 5′- GTGGAATTGATGGCGGGTCCTGAGGGTGCGGGTCTCAGTATAATTG -3′ and 5′- CAATTATACTGAGACCCGCACC CTCAGGACCCGCCATCAA TTCCAC -3′.

### Clonings for crystal trials, ITC and GST pull-down assays

The constructs comprising the PDZ domains of *dm*Spn (residue 1,258–1,347), *dm*Syd-1 (residue 155–242) and *rn*Spn (residue 493–583) were amplified by PCR and cloned into the pET-MBP vector using NcoI and SalI restriction sites with primers: *dm*Spn_fwd: 5′- TATACCATGGCGCATGTCTTCCCCGTGG -3′, *dm*Spn_rev: 5′- TATA CCATGGTGGCCGCTTCGG -3′, *dm*Syd-1_fwd:5′- TATACCATGGCGCAGGCGGTCGATGC -3′, *dm*Syd-1_rev:5′- TATACCATGGCGCACACGGTTCAACTTGTCG -3′, rnSpn_fwd: 5-′ TATACCATGGAGCTGTTTCCTGTGGAG -3′ and rnSpn_rev: 5′- ATATGTCGACCTACTCCCGGCCAATCATG -3′.

The resulting constructs contained an N-terminal His6-MBP-tag followed by a tobacco etch virus cleavage site and the respective PDZ domain. The constructs comprising the last 10 C-terminal amino acids of dmNrx-1 (residue 1,831–1,840) and rnNrx-1 (residue 1,498–1,507) were amplified by PCR and cloned into the pGEX-6-P1 vector by a SLIC reaction using overlapping primers: dmNrx-1ct_fwd: 5′- GACTCCAAGGACGTCAAGGAGTGGTATG TGTAACTGACGATCTGCCTCG -3′, *dm*Nrx-1 ct_rev: 5′- TTACACATACCACTCCTTGACGTC CTTGG AGTC GTCACGATGCGGCC -3′, *r*Nrx-ct_fwd: 5′- AAGAAGAACAAAGACAAAGAGTATTACGTCTAGCTG ACGATCTGCCTCG -3′, *r*Nrx-1ct_rev: 5′- CTAGACGTAATACTCTTTGTCTTTGTTCTTCTTGTCAC GA TGCGGCC-3′.

The resulting constructs comprised an N-terminal GST-tag followed by a PreScission cleavage site and the respective 10 C-terminal amino acids of Nrx-1. Detailed version of methods for Protein expression and purification, ITC assays and crystallization are presented in Supplementary Methods.

### Generation of Spn genomic constructs

Pac (Spn^1^) was created from P[acman] BAC clone CH321-01N11 (genomic region 2499270 to 2581398; CHORI-321 library of the BACPAC Resource Centre), which was subjected to transgenesis using the Phi31 system (P[acman] strain 24872, M[vas-int.Dm]ZH-2A, PBac[y[+]-attP- 3B]VK00037). Similarly, Pac(Spn^2^) was obtained by injecting the P[acman] BAC clone CH321-67O06 (genomic region 2469714 to 2556468). Pac(Spn*) corresponds to P[acman] BAC clone CH321-67O06, but lacks the whole Spn open reading frame, and was cloned according to the Counter Selection BAC Modification kit obtained from Gene Bridges GmbH. rpsL-neomycin (neo) template DNA was used to generate selectable cassettes. Primers contained a 50-bp homology region and a sequence for amplification of the rpsL-neo counter selection cassette. Selectable cassettes were generated by PCR using Vent Polymerase (New England Biolabs, Inc.) and the following primer pairs. Spn-rpsL-fwd:5′- GGCCCGAAATTCAAGCTAAACGGACGCGTTTTCGTCGCGAGTTTAACC GCGGCCTGGTGATGATGGCGGGATCG -3′, Spn-rpsL-rev: 5′- ATTTCAGAGTATATTTATTAGCACTGATTTTGAGATTTATT ATTTTCCATTCAGAAGAACTCGTCAAGAAGGCG -3′.

### Yeast-2-hybrid clones

Yeast-2-hybrid analysis was carried out using the LexA system (pB27 bait vector; pP6 bait vector). The cytoplasmic C terminus of Nrx-1 was cloned into pB27 using primers: 5′- GATGGAATTC-AATGGCGATCGTGGCT -3′ and 5′- GTCTATACTAGT-TTACACATACCACTCCTTGACGTCCT -3′.

The Spn and Syd-1 fragments depicted in Fig. 6 were cloned into pP6 using: F1-fwd: 5′- CAATTCCATGGC-CATGGAGAAACCGATGCATCAT -3′, F1-rev: 5′- CAACCTCGAGTTA-ATA GC CGACGTCCACGTA -3′, F2-fwd: 5′- CAAACCATGGCC-GGTCGCAAATCTGTGGACG -3′, F2-rev: 5′- CTTGGATCCTT-ACTCGTGCAGTGATTCCCC -3′, F3-fwd: 5′- GATCCATGGCC-CGTGAAGAGCTGGAAAAC -3′, F3-rev: 5′- GTTGGATCCTTA-CGTCTTACGCATCATCTG -3′, F4-fwd: 5′- GATCccatggccGAGGAGCGCTTGAAGCGCCAA -3′, F4-rev: 5′- CTGGGATCCTTGTGCACCTGGGCATA -3′, F5-fwd: 5′- GATC CCATGGCCAACTCGCATCTGCTGGCCAACGTG -3′, F5-rev: 5′- GGAATCCTCGAG-CTTCTTTTTGGCCGCCTTCTTCT -3′, Syd-1 F1-fwd: 5′- GTCTATGAATTC ATGACG GTGC AACC GGCTGAA -3′, Syd-1 F1-rev: 5′- GTCTATACT-AGTT CCCGTT GACATTC TTCTCG -3′.

### Immunostaining and imaging

Larval filets were dissected and stained as described previously^13^,^21^. Primary antibodies used were: rabbit (Rb) SPN^N2.2^ (1:3,000), RbGluRIID (1:500), RbDSyd-1 (1:500), RbNlg1 (1:500), RbDRBP (1:500) and guinea pig Nrx-1 (1:500) (generously provided by M. Bhat). We used MNc82 (1:100) and MCSP (1:500) (Developmental Studies Hybridoma Bank (DSHB), the University of Iowa, Iowa City, IA), MFasII (1D4; DSHB), mouse monoclonal antibody 3E6 (to stain GFP) (1:500) (Invitrogen) and rabbit anti-dsRed (1:500) (Clontech). Secondary antibodies were generally diluted 1:500. Secondary antibodies for STED were used in the following concentrations: goat anti-mouse Atto590 1:100 and goat anti-rabbit star635 1:100. The dyes Atto590 (ATTO-TEC) and Star635 (Abberior) were coupled to the stated IgGs (Dianova). Imaging larvae were mounted in Mowiol (Sigma-Aldrich) for STED.

The sizes and surface densities of AZ cluster (visualized using BRP^nc82^, RimBP and Cac^GFP^) were quantified from maximal projections of confocal NMJ stacks. A Cy5-HRP antibody (23-175-021, Jackson ImmunoResearch, 1:250) was used to outline the shape of the NMJ. Control and mutant larvae were stained in the same vial. All images for synapse quantification from fixed samples were acquired using the same microscope settings (with × 63 magnification and numerical aperture 1.4 oil objective, Leica). AZ cluster analysis was done as described previously^69^; AZ densities were obtained by normalizing the total number of particles analysed to the total synaptic area (pixel units) measured via HRP. Similarly, the absolute intensities of synaptic proteins per NMJ were normalized to the absolute intensity of synaptic HRP of the corresponding NMJ.

### *In vivo* imaging and FRAP analysis

All UAS constructs were driven in motoneurons using OK6-Gal4^75^. Intravital live imaging was performed as described previously^13^,^21^.

### STED and EM

STED microscopy was performed as described previously^30^. BRP ring diameter measurements were performed on deconvolved images. Line profiles were placed across the middle of planer-oriented BRP rings and the longest peak-to-peak distance measured. Five to seven images obtained from four to five third instar larvae per genotype were processed and analysed.

### Head fractionation, co-immunoprecipitation and Y2H assay

We followed a new protocol using *Drosophila* head fractionation, to obtain protein extracts used in co-immunoprecipitation experiments. Extracts were run on 6% Tris_HCl gels. Proteins were then transferred onto a nitrocellulose membrane and blocked with 5% milk in 1 × PBS supplemented with 0.1% Tween-20 (PBS-T). Membranes were probed with guinea pig anti-Nrx-1 (1:5,000; a custom polyclonal directed against the last 100 amino acids of Nrx-1) and rabbit anti-Spn^N2.2^ (1:10,000) diluted in PBS-T. After washing, secondary anti-guinea pig or anti-rabbit HRP-conjugated antibodies were used for detection (Dianova) in conjunction with an enhanced chemoluminescence (GE Healthcare ECL Prime; product number RPN 2232) detection system with Hyperfilm ECL (GE Healthcare). Films were scanned in transmission mode (Epson V770). Images were imported to Photoshop (Adobe), and brightness and contrast were adjusted. The liquid Y2H *β*-galactosidase assay was performed as reported previously^70^.

### Co-immunoprecipitation from mouse brain

Brains were homogenized in 25 ml per g tissue in homogenization buffer (50 mM Tris-HCL, pH7.4, 150 mM NaCl, 10% glycerol, 2 mM caCl2+EDTA free protease and phosphatase inhibitor mixes) using glass homogenizer. After homogenization samples were sonicated with 3 × 10 pulses, Triton-X100 was added to the final concentration of 1% and homogenate was incubated for 10 min at 4 °C with rotation. Sample was sonicated again with 10 pulses. Samples were spun down at 20,000 × *g* for 30 min. About 10 μl per ml homogenate of protein A/G magnetic beads were added following 30 min incubation and separation of magnetic beads from the homogenate. Homogenate was aliquoted in 2 ml tubes (1.6 ml per tube) and 0.8 μg affinity-purified anti-pan-NRX or rabbit IgG was added to each aliquot. Samples were incubated overnight with rotation at 4 °C. About 8 μl protein-A magnetic beads (Dynabeads) were added and samples were incubated for additional 2 h. Samples were washed 3 × with homogenization buffer+0.1% Triton-X100 and once with homogenization buffer without detergent. Bound proteins were eluted with 30 μl 2% sodium deoxycholate. Eluted proteins were separated on 8% PAA gel and probed with anti-spinophilin (1:1,000, Cell Signaling, E1E7R) and anti-pan-Nrx (40 μg ml^−1^, homemade, affinity purified).

### Two-electrode voltage clamp recordings

TEVC recordings were performed on larval NMJs of third instar males (muscle 6 and segments A2 and A3), essentially as described^6^. The composition of the extracellular hemolymph-like saline (HL-3) was (in mM) NaCl 70, KCl 5, MgCl2 20, NaHCO_3_ 10, trehalose 5, sucrose 115, HEPES 5 and CaCl2 1.5, pH adjusted to 7.2. Recordings were made from cells with an initial membrane potential (*V*m) between −50 and −70 mV and input resistances of ⩾4 MΩ, using intracellular electrodes with resistances of 8–20 MΩ and filled with 3 M KCl. eEJCs, which reflect the compound excitatory junctional current of both the motoneurons innervating muscle 6 (voltage clamp at −60 mV) and mEJCs (voltage clamp at −80 mV) were low pass filtered at 1 kHz. The 0.2-Hz stimulation protocols included 20 traces per cell. Paired-pulse recordings consisted of 10 traces per interval per cell in which a 4-s rest was left between paired pulses. For determination of the base line of the second pulse at the 10-ms interpulse interval, the decay of the first pulse was extrapolated. Recordings were analysed with pClamp 10 (Molecular Devices). Stimulation artifacts in eEJC recordings were removed for clarity.

### GCaMP5 imaging; assaying spontaneous and evoked release by Ca^2+^ imaging

Optical analysis of spontaneous and evoked transmitter release was performed similarly as described^3^ by imaging postsynaptic GCaMP5 fluorescence signals in flies expressing UAS-myrGCaMP5. Local activity patterns were aligned to confocal images of a post-fixed staining against GFP and BRP to identify single AZs. See Supplementary Methods for full details of Ca^2+^ imaging, image alignment and signal processing.

### Statistics

Data were analysed using Prism (GraphPad Software). Nonparametric Mann–Whitney *U*-tests were used to compare two groups for all data sets. Nonparametric Kruskal–Wallis tests were used for comparison of more than two groups, followed by a Dunn's multiple comparison test. *P* values, *n* values and *U* or *K* statistics are given in the figure legends or main text. Similarly, the electrophysiological data are reported as mean±s.e.m. and *P* value denotes the significance according to one-way analysis of variance with Tukey's multiple comparison post-test.

## Additional information

**Author Information.** The structure factors and atomic coordinates of the Spn-PDZ domain are deposited in the Protein Data Bank with accession number 4XHV.

**How to cite this article:** Muhammad, K. G. H. *et al*. Presynaptic spinophilin tunes neurexin signalling to control active zone architecture and function. *Nat. Commun.* 6:8362 doi: 10.1038/ncomms9362 (2015).

## Supplementary Material

Supplementary Information Supplementary Figures 1-12, Supplementary Table 1-2, Supplementary Methods and Supplementary References

Supplementary Movie 1 Live movie of GCaMP5 fluorescence during 98 s of spontaneous activity at Control NMJs

Supplementary Movie 2 Live movie of GCaMP5 fluorescence during 35 episodes of AP stimulation at Control NMJs

Supplementary Movie 3 Live movie of GCaMP5 fluorescence during 98 s of spontaneous activity at Spn NMJs

Supplementary Movie 4 Live movie of GCaMP5 fluorescence during 35 episodes of AP stimulation at Spn NMJs
