# Supplementary material for: Presynaptic spinophilin tunes neurexin signalling to control active zone architecture and function
Source: Nat Commun. 2015 Oct 16;6:8362. doi: 10.1038/ncomms9362 (PMC4633989; doi:10.1038/ncomms9362)
Supplement: Supplementary Information — Supplementary Figures 1-12, Supplementary Table 1-2, Supplementary Methods and Supplementary References [file ncomms9362-s1.pdf]

Supplementary Figure 1

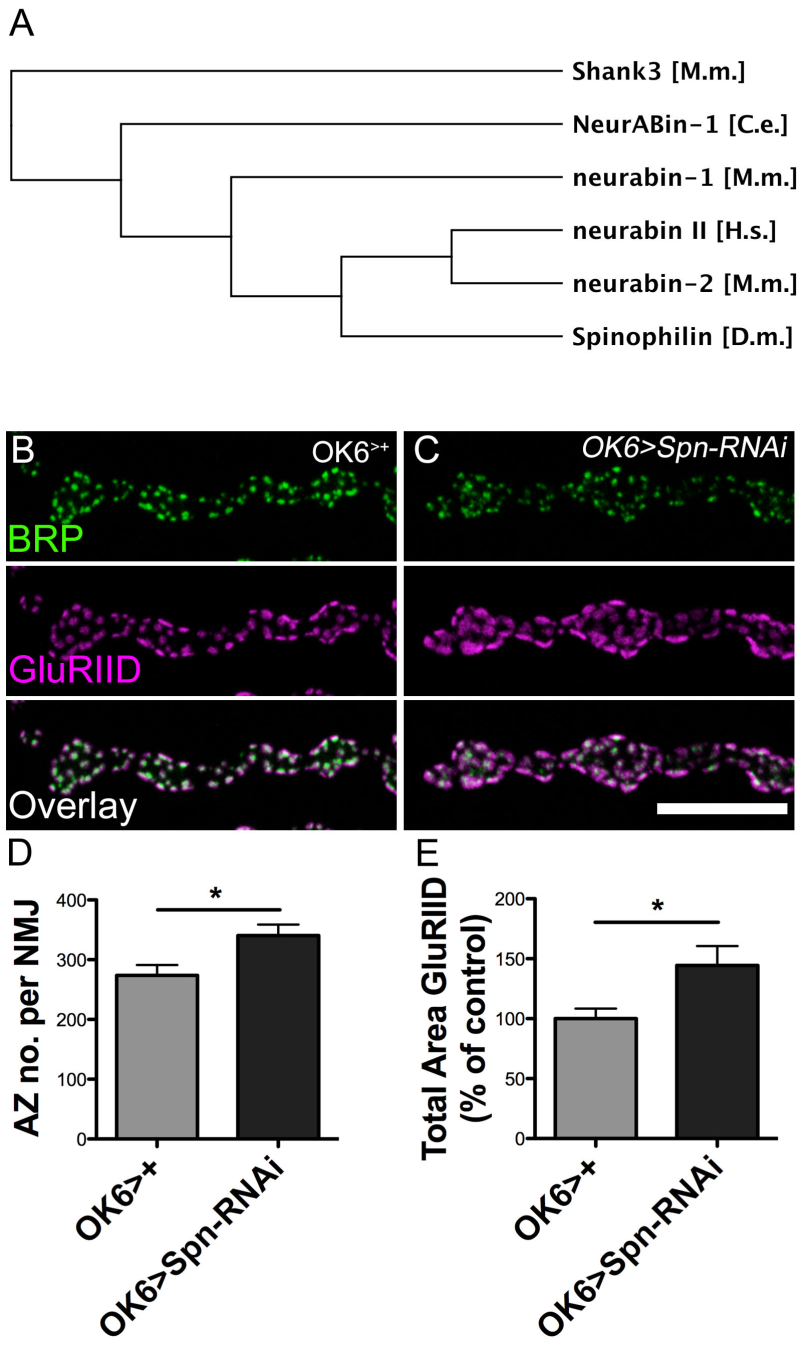

Supplementary Figure 1 related to figure 1: (**A**) Dendrogram analysis comparing Neurabin family of proteins together with that of Drosophila Spn. Shank3 has been used as an out-group. **(B-C)** RNAi mediated knockdown of presynaptic Spn results in more AZ scaffolds and enlarged glutamate receptor field size. **(D-E)** Quantifications of total BRP spots and Glutamate receptor field sizes in motoneuron derived Spn-RNAi, BRP spots: in ctrl : 274± 17.4, n = 9; *Spn*: 340 ±18.1 , n = 10; ctrl versus *Spn* P< 0.05 ; Mann-Whitney U-test (U = 15).Total GluRIID area: in ctrl: 77.8± 6.5, n = 9; *Spn*: 113.6 ±11.2 , n = 10; ctrl versus *Spn* P<0.05 ; Mann-Whitney U-test (U = 17). Scale bar 10µm.

Supplementary Figure 2

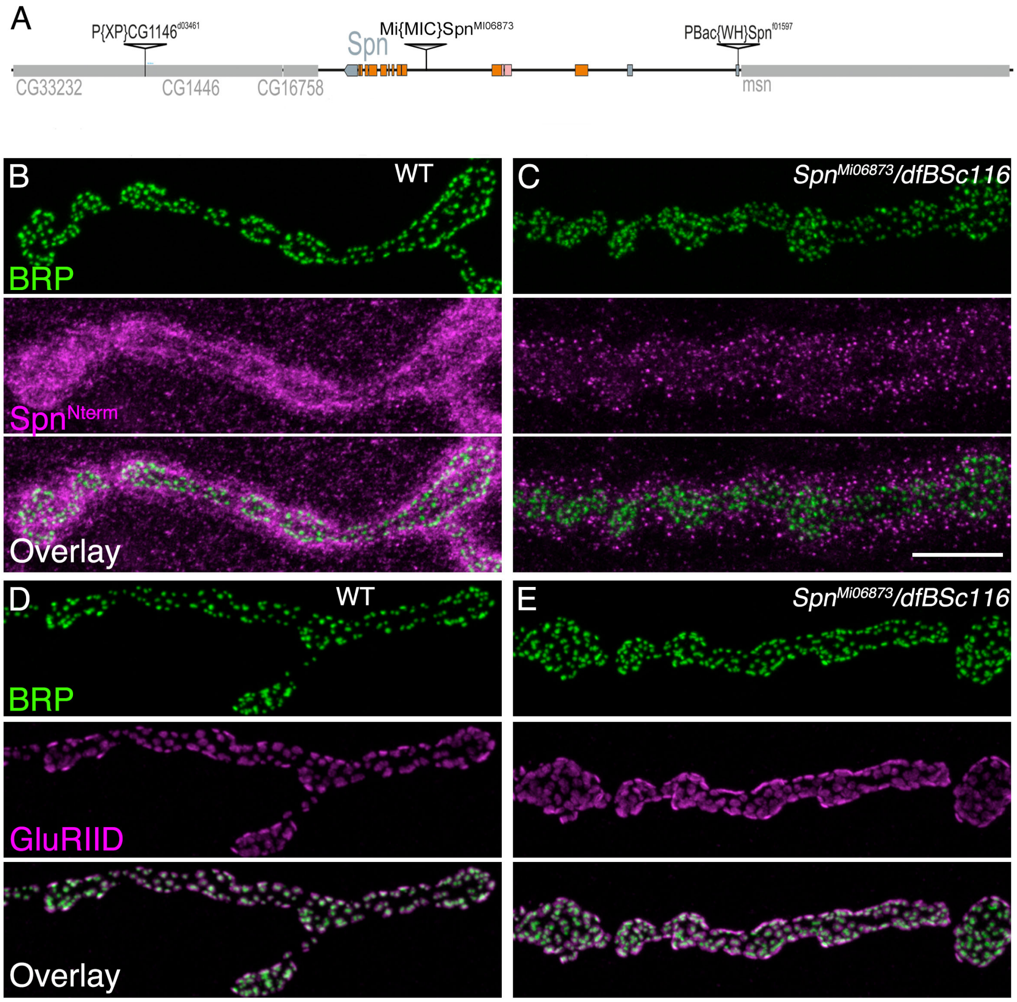

Supplementary Figure 2 related to Figures 1&2. Additional allele of *spn* loucs, MiMic-Spn^{Mi06873} shows similar phenotypes of *Spn* larvae. **(A)** Genomic locus of transposon element integration. **(B-C)** Reduced anti-Spn staining at the NMJ of Spn^{Mi06873} allele with concomitant increase of BRP spot density. **(D-E)** Enlarged GluRIID field size in MiMic-Spn^{Mi06873} hypomorphic allele. Scale bar 10 µm.

Supplementary Figure 3

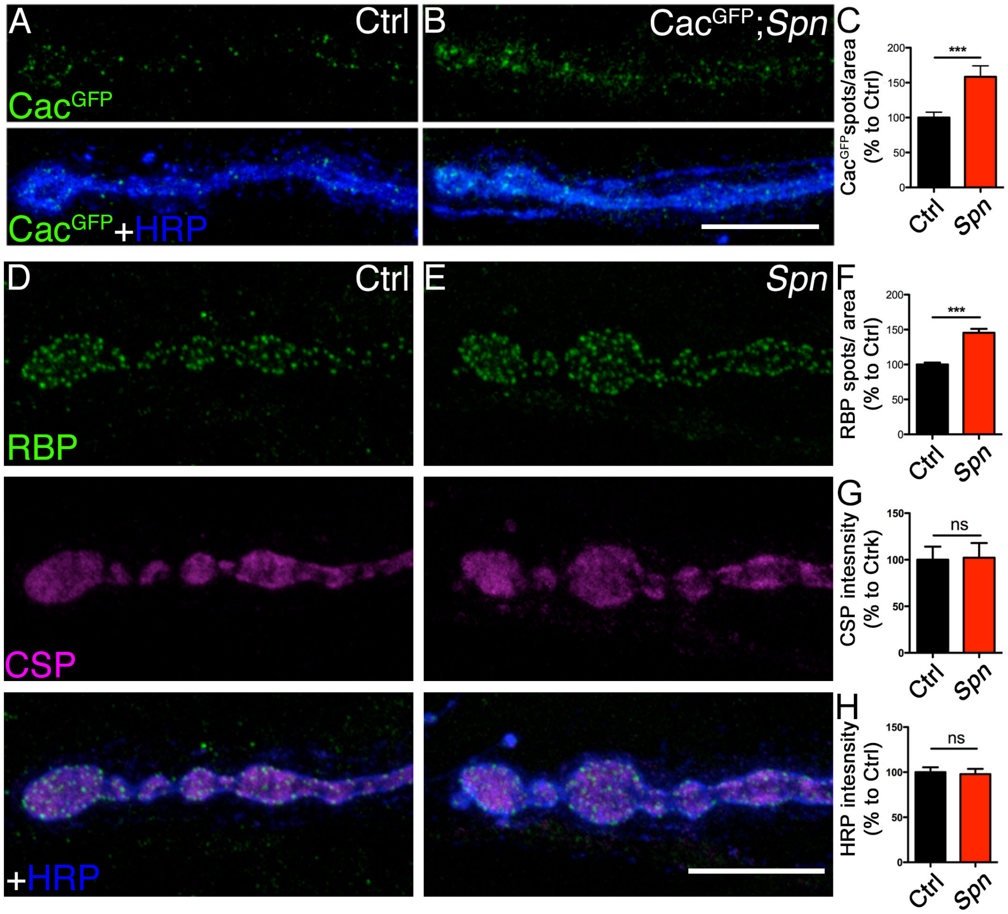

Supplementary Figure 3 related to Figures 2 and 3. RBP and Cac^{GFP} spot density increased at *Spn* NMJs. **(A)** UAS-Cac^{GFP} expression in Ctrl compared to **(B)** *Spn* show significant increase of total amount of Ca^{2+} channels at *Spn* NMJs. **(D-E)** RBP spot density is similarly increased in *Spn* NMJ while synaptic vesicle marcker CSP does not alter. **(C, F-H)** Cac^{GFP} in Ctrl: 100 ± 7.6, *n* = 9; Cac^{GFP} in *Spn*: 158 ± 15.67 *n* = 8; Ctrl versus *Spn* P<0.001 Mann-Whitney U-test (U = 4), (RBP in Ctrl: 100 ± 2.7, 771 *n* = 10; RBP in *Spn*: 145.5 ± 5.6 *n* = 10; Ctrl versus *Spn* P<0.001 Mann-Whitney U-test (U = 1), (CSP in Ctrl: 100 ± 14, *n* = 10; CSP in *Spn*: 102 ± 15.64 *n*= 10; Ctrl versus *Spn* P>0.05 Mann-Whitney U-test (U = 47), (HRP integrated intensity in Ctrl: 100±5.4, n=10; HRP in Spn: 97.8±5.9, n=10; Ctrl versus *Spn* P>0.05 Mann-Whitney U-test (U = 41). Error bars indicate SEM. *, P < 0.05; **, P < 0.01; ***, P < 0.005; ns, P > 0.05. Scale bar: 10μm.

Supplementary Figure 4

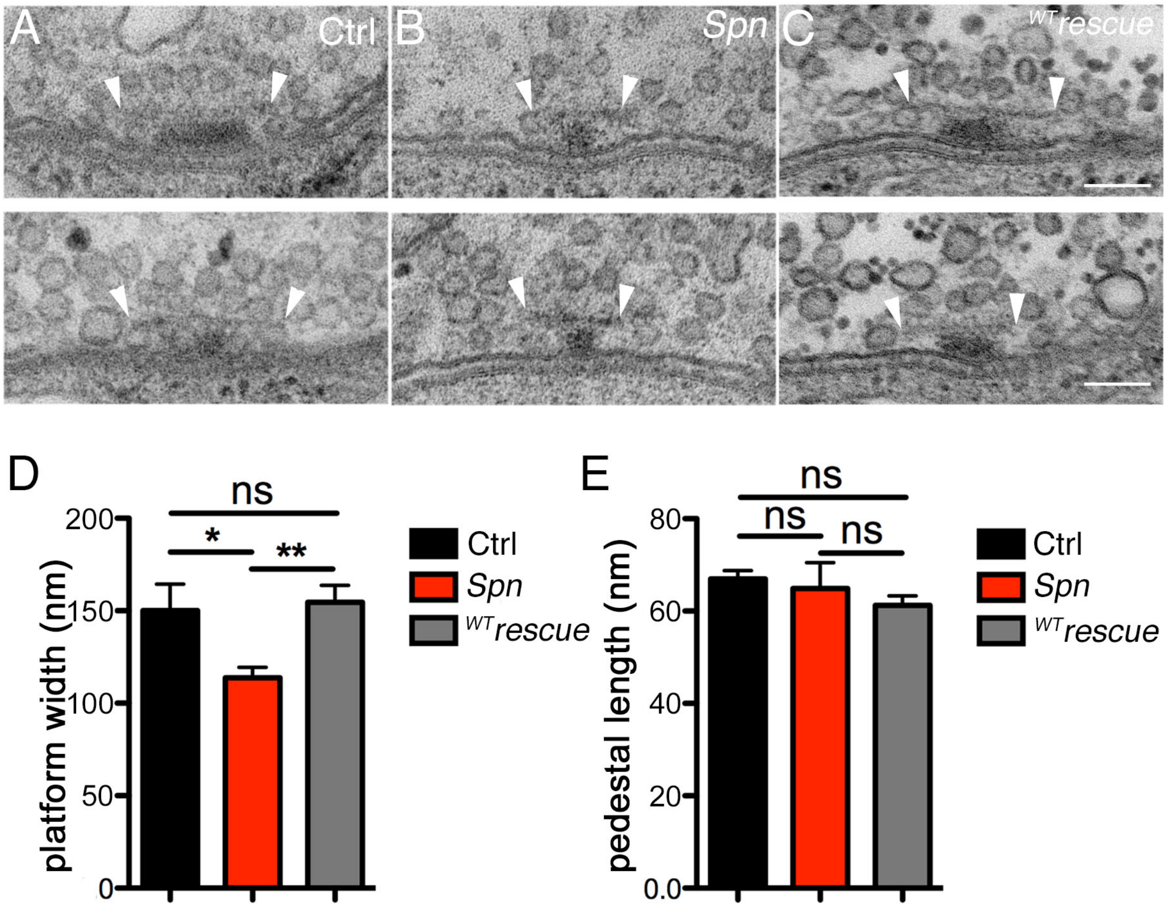

Supplementary Figure 4 related to Fig.3. Electron dense projection analyses in *Spn* NMJs. Two independent examples of dense projection (T-bars) are shown per indicated genotypes of Ctrl **(A)**, *Spn* **(B)** and ^{WT}Spn rescue**(C)**. **(D)**T-bar platform's width (electron-dense materials parallel to double membrane) were significantly shorter in *Spn* compared with Ctrl and ^{WT}rescue (Ctrl: 150±14.3 nm, n=12; *Spn*: 113.8 ± 5.6 nm, P<0.05, n=21; ^{WT}Spn-rescue: 154± 9 nm, P<0.01 n=19; one-way ANOVA Tukey's posttest). **(E)** The T-bar height (pedestal plus platform perpendicular to double membrane) was unaffected in *Spn* compared to controls (Ctrl: 66.9±1.7 nm, n=1; *Spn*: 64.8±5.6 nm, P>0.05, n=21; ^{WT}Spn-rescue: 61.3±2.08 nm, P> 0.05, n=19; one-way ANOVA Tukey's posttest). All panels show mean values and errors bars representing SEMs. *, P ≤ 0.05; n.s., P > 0.05. Scale bar: 100 nm.

Supplementary Figure 5

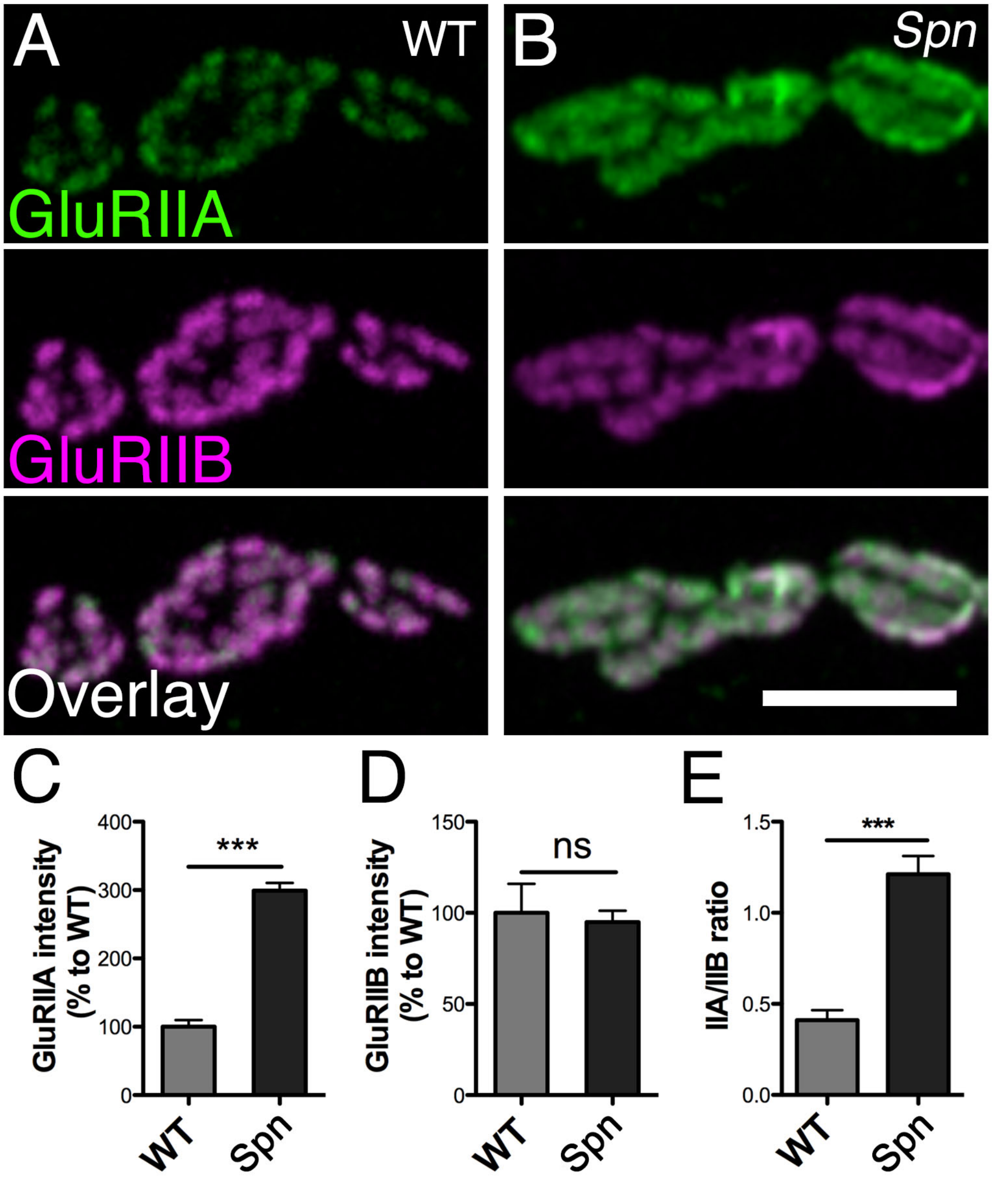

Supplementary Figure 5 related to Fig.4. Spn controls postsynaptic GluR field size and composition. **(A-B)** Co-labelling of DGluRIIA and DGluRIIB for wild type **(A)**, *Spn* **(B)**, NMJs. **(C)** Integrated GluRIIA signal (wild type: 100±9.9, n=7; Spn: 299.3±11.15, n=8;WT versus *Spn* *P*<0.001; Mann-Whitney *U*-test (*U* = 0.0). **(D)** Integrated GluRIIB signal (wild type: 100±15.9, n=7; Spn: 94.95±6.2, n=8; WT versus *Spn* *P:* 0.8 ; Mann-Whitney *U*-test (*U* = 26). **(E)** Increased incorporation of GluRIIA compared to GluRIIB (wild type: 0.41±0.05, n=7; *Spn*: 1.2±0.1, n=8; wild type versus *Spn* *P*< 0.001; Mann-Whitney *U*-test (*U* = 1). Scale bar 5 µm.

Supplementary Figure 6

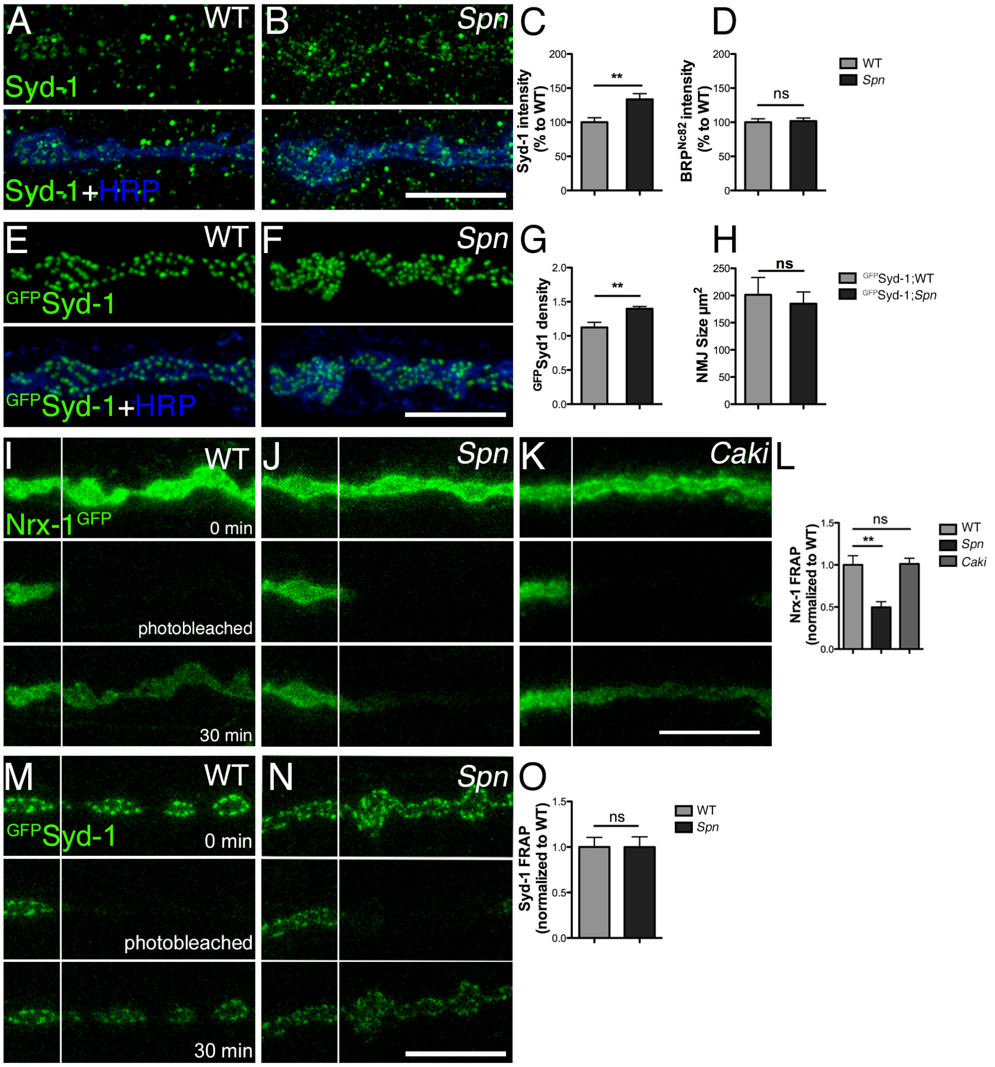

Supplementary Figure 6 related to Fig.4. Elevated levels of endogenous Syd-1 in *Spn* NMJs. **(A)** co-labelling of Syd1 and HRP in WT and **(B)** in *Spn* NMJs. **(C)** Total amount of Syd-1 is increased in *Spn*, Wild type: 100± 6, n = 14; *Spn*: 133.5 ±8.4, n = 19; wild type versus *Spn* $P<0.01$; Mann-Whitney U-test (U = 55). **(D)** while amount of BRP is unaffected, Wild type: 100± 5, n =14; *Spn*: 101.8 ±4.5 , n = 19; wild type versus *Spn* $P> 0.05$; Mann-Whitney U-test (U = 123). The NMJ size (chart not depicted), measured via HRP staining, is not changed in *Spn*, Wild type: 228± 11, n = 14; *Spn*: 206 ±7.7 , n = 19; wild type versus *Spn* $P> 0.05$; Mann-Whitney U-test (U = **(E-H)** Similarly, ^{GFP}Syd-1 over-expression in *Spn* **(F)** shows more but smaller ^{GFP}Syd1 spots compared to controls **(E)**. Quantifications of ^{GFP}Syd-1 spot

density **(G)** and NMJ size **(H)** in control and *Spn* animals, ^{GFP}Syd-1 spots: Wild type: 1.12± 0.07, *n* = 7; *Spn*: 1.39 ±0.03 , *n* = 6; wild type versus *Spn* *P*<0.01 ; Mann-Whitney *U*-test (*U* = 2). NMJ size: wild type: 201.4±31.8, n=7; Spn: 184.8±21.6 μm^2; wild type versus *Spn* *P*>0.05 ; Mann-Whitney *U*-test (*U* = 18). **(I-L)** FRAP of Nrx-1^{GFP} expressed in motor neurons of wild type, *Spn* and *Caki* larvae. The middle (photobleached) rows were taken 2 min after the top row. (**L**) Quantifications of the Nrx-1^{GFP} recovery signal in respected genotypes were normalized to its recovery in wild type larvae. Nrx-1^{GFP} in wild type: 1.0±0.1, n=47; Nrx-1^{GFP} in *Spn* :0.49±0.06, n=14; Nrx-1GFP in *Caki*: 1.01±0.06, n=27. Nrx-1^{GFP} recovery in wild type versus *Spn*, P<0.001 Mann-Whitney *U*-test (*U* = 138), Nrx-1^{GFP} recovery in wild type versus *Caki*, P>0.05 Mann-Whitney *U*-test (*U* = 518). **(M-O)** FRAP of ^{GFP}Syd-1 expressed in motor neurons of wild type and *Spn* larvae. The middle (photobleached) row taken 2 min after the top row. (**O**) Quantifications of ^{GFP}Syd-1 recovery signal normalized to wild type. ^{GFP}Syd-1 in wild type: 1.0 ±0.1, *n* = 9; ^{GFP}Syd-1 in *Spn* :1.0±0.1, *n* = 6; ^{GFP}Syd-1 in wild type versus *Spn* background, *P>0.05*; Mann-Whitney *U*-test (*U* = 27). Scale bars 10µm.

Supplementary Figure 7

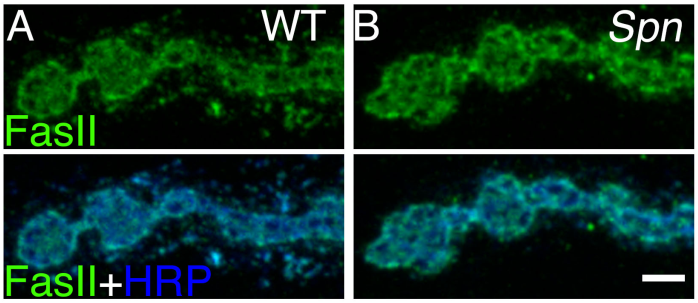

Supplementary Figure 7 related to figure 4 Synaptic cell adhesion molecule Fasciclin II staining in *Spn* NMJs. Co-labelling of FasII staining with HRP marker in (A) wild type and **(B)** *Spn* NMJs, show no changes upon lack of Spn. Scale bar 2.5 µm.

Supplementary Figure 8

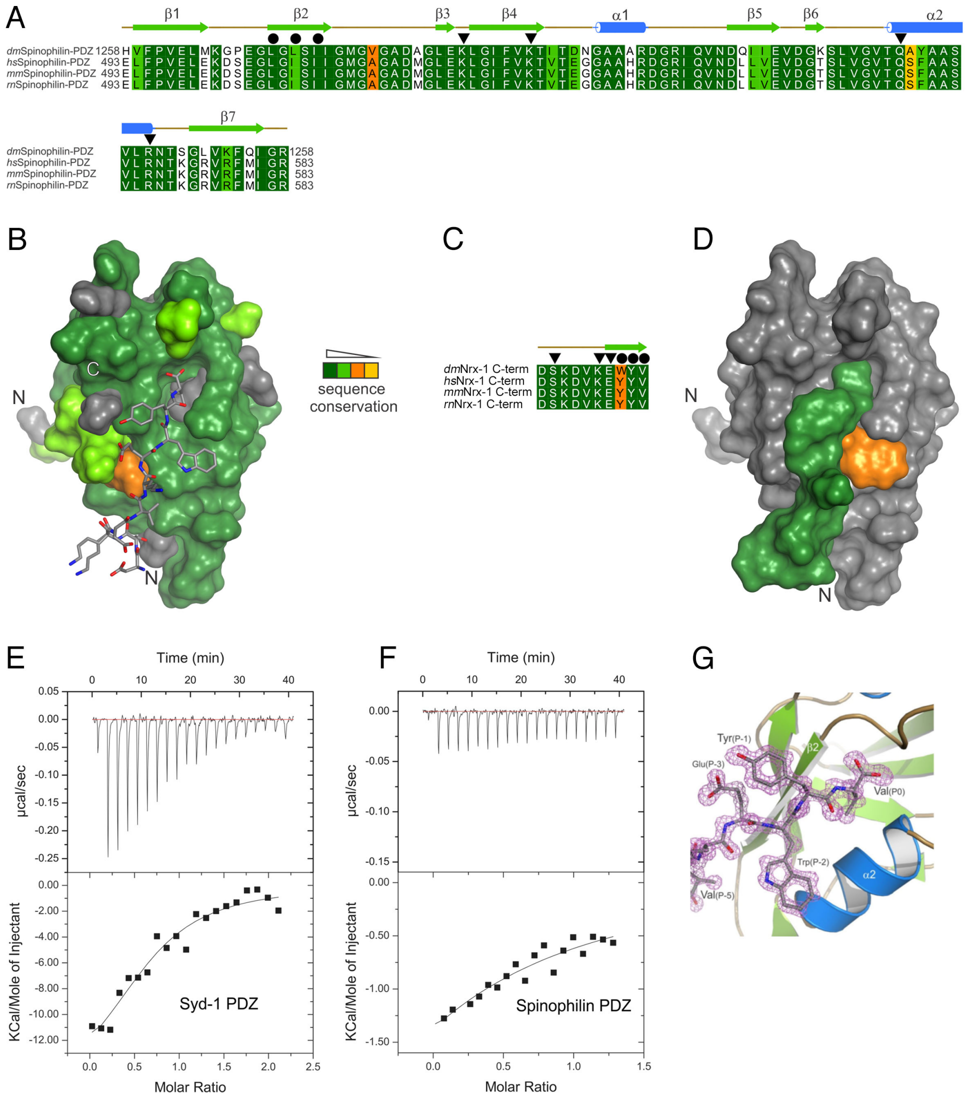

Supplementary Figure 8 related to figure 5. Similarity between Nrx-1 C-termini and PDZ domains of fly and rodent animal models. **(A)** Alignment of PDZ domains from *dm*Spinophilin, *Homo sapiens* Spinophilin (*hs*Spinophilin), *Mus musculus* Spinophilin (*mm*Spinophilin), and *Rattus norvegicus* Spinophilin (*rn*Spinophilin). Secondary structure elements are indicated on top of the sequences. Filled circles indicate residues involved in *dm*Spinophilin protein backbone to peptide backbone interactions and triangles describe residues involved in side chain interactions. **(B)** Sequence conservation is mapped on the surface of the crystal structure of *dm*Spinophilin-PDZ. The bound *dm*Neurexin peptide is shown in stick representation. Secondary structure elements are indicated on top of the sequences. Filled circles indicate residues involved in *dm*Spinophilin protein backbone to peptide backbone interactions and triangles describe residues involved in side chain interactions. **(C)** Alignment of the last ten C-terminal amino acid residues of *dm*Neurexin, *Homo sapiens* Neurexin (*hs*Neurexin), *Mus musculus* Neurexin (*mm*Neurexin), and *Rattus norvegicus* Neurexin (*rn*Neurexin). (**D**) Sequence conservation is mapped on the surface of the bound *dm*Neurexin peptide. *dm*Spinophilin-PDZ is

shown as gray surface. **(E,F)** Quantification of protein-peptide interactions by ITC. Both the raw data and the integrated data are shown. Data were fitted based on the “One Set of Sites” model. (**E**) Titration of MBP-*dm*Syd-1 PDZ and the *dm*Neurexin peptide. In a control experiment with MBP, we could not detect any binding of the *dm*Neurexin peptide. (**F**) Titration of *dm*Spinophilin-PDZ and the *dm*Neurexin peptide. (**G**) m*F*oD*F*c simulated annealing omit map shown as violet mesh contoured at 3.0 σ around the bound peptide. For calculation of the electron density map the *dm*Neurexin peptide had been omitted. The peptide is shown in gray stick representation and *dm*Spinophilin-PDZ in cartoon representation.

Supplementary Figure 9

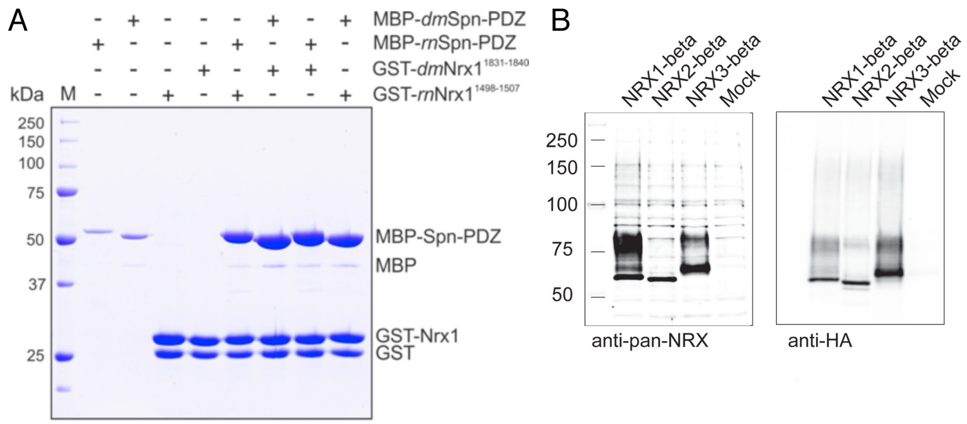

Supplementary Figure 9 related to Fig. 5 **(A)** MBP-*dm*Spinophilin (MBP-*dm*Spn PDZ) and MBP-*rn*Spinophilin (MBP-*rn*Spn PDZ) pulldown assays in the presence of GST- *dm*Neurexin1 (GST-*dm*Nrx1$^{1831\text{-}1840}$) and GST-*rn*Neurexin 1 (GST-*rn*Nrx1$^{1498\text{-}1507}$) peptides and the indicated proteins. **(B)** Immunoprecipitation of Nrx-1 complex from mouse brain homogenate.

Supplementary Figure 10

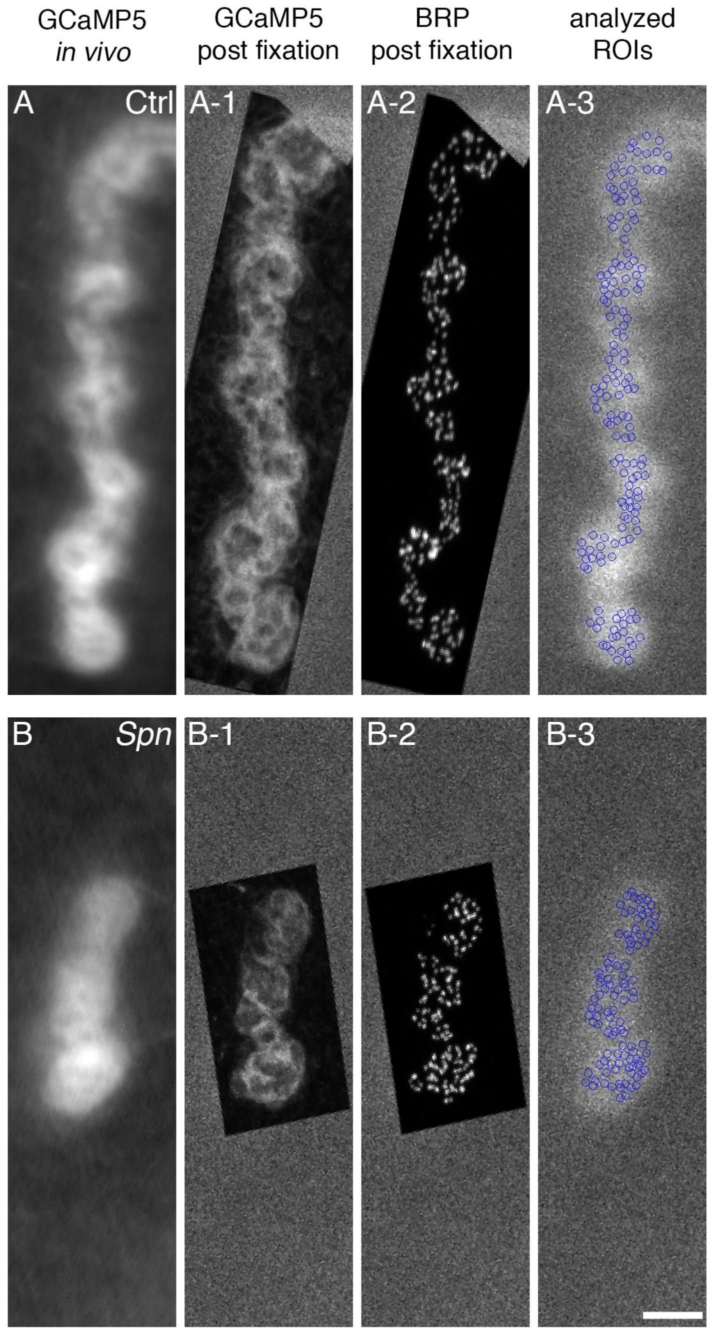

Supplementary Figure 10 related to figure 7 Assignment of single active zones identified by post-hoc staining against Bruchpilot (BRP) to GCaMP5 events at control and *Spn* NMJs. **(A, B)** *In vivo* GCaMP5 signal, produced from live movies by an average projection of 2000 frames acquired during spontaneous activity. **(A1, B1)** GCaMP5 signal after fixation, shown is a Z-projection of confocal light microscopic scans. Individual areas of the confocal image were registered to the first frame of the *in vivo* GCaMP5 signal (see Methods). A2, B2) The same registration transformation as for the confocal GCaMP5 images (A1, B2) was used to align individual active zones identified in confocal scans by staining against the presynaptic active zone marker BRP. **(A3, B3)** BRP

staining was used to place uniformly sized regions of interest (ROIs) to read out GCaMP5 fluorescence over time. Images A1, A2, A3 and B1, B2, B3 are placed on top of the first frame acquired in the live Ca^{2+}-imaging experiment (exposure time 0.05s). Scale bar 5μm.

Supplementary Figure 11

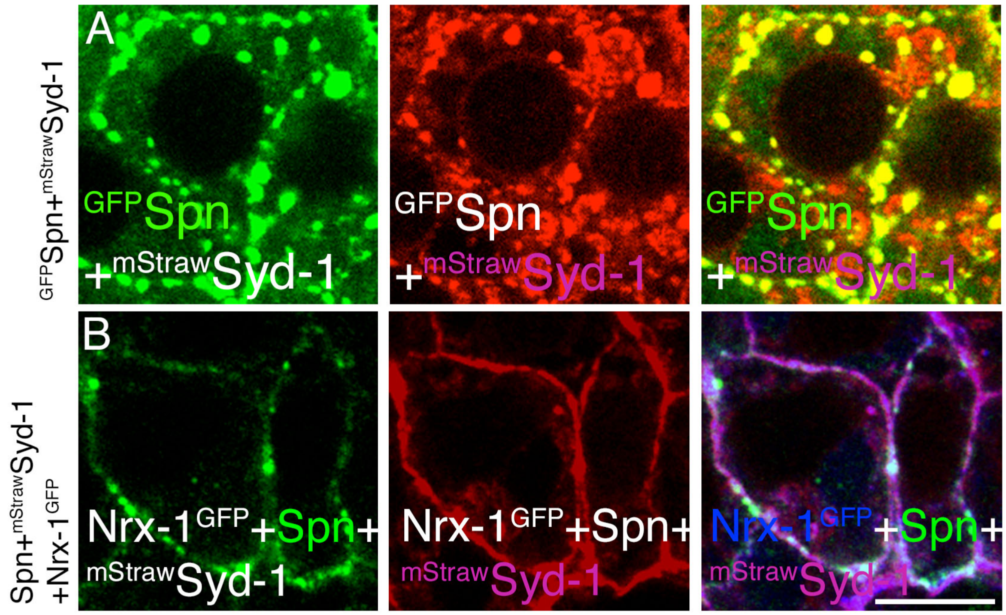

Supplementary Figure 11 In-vivo complex formation between ^{GFP}Spn, ^{mStrawSyd}-1 and Nrx-1^{GFP}. **(A)** co-expression of ^{GFP}Spn and ^{mStraw}Syd-1 leads to co-aggregation of both proteins at the membrane but also in cytoplasm of salivary gland cells. **(B)** Addition of Nrx-1^{GFP} to **(A)**, triple expression, leads to diffused localization of both Spn and Syd-1 at the membrane. Scale bar 25μm.

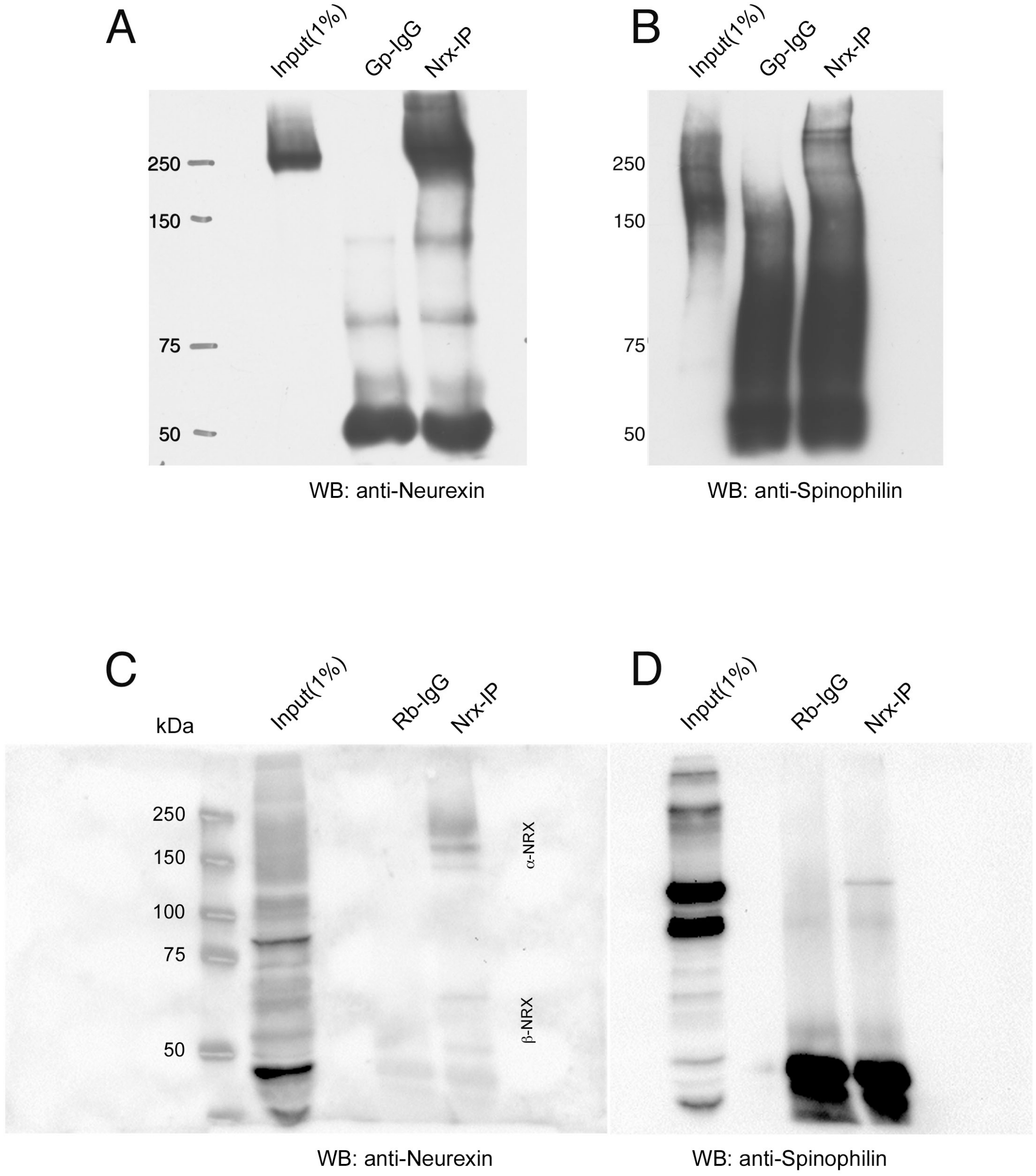

Supplimentary Figure 12 Conserved interaction between Spn and Nrx-1 as shown in Fig.5. **(A-B)** Immunoblot (un-cropped) of fly Nrx-1 immunoprecipitate (IP) from Drosophila head fractionation sample enriched for AZ proteins (see Methods). **(A)** Enrichment of Nrx-1 in ginue pig anti-Nrx-1 Co-IP sample. **(B)** Spn bands of expected size can be detected in Nrx-1 Co-IP sample, but is absent in control immunoglobulin G is used (IgG). **(C-D)** Similary this interaction could be detected in rodent brain homogenates. **(C)** Western-blot analysis of a pan-Nrx antibody IP. **(D)** Co-IP complex with anti-Spn antibody.

Supplementary Table 1 Data collection and refinement statistics for *dm*Spinophilin-PDZ bound to a *dm*Neurexin derived peptide.

**Data collection**	
PDB entry	4XHV
Space group	$P4_32_12$
Wavelength [Å]	0.91841
Unit cell a; b; c [Å]	45.3; 45.3; 94.5
α; β; γ [º]	90.0; 90.0; 90.0
Resolution [Å] [a]	45.30-1.23 (1.30-1.23)
Unique reflections	29395 (4571)
Completeness [a]	99.7 (98.5)
$<I/\sigma(I)>$ [a]	21.6 (2.4)
R_{meas} [a, b]	0.060 (0.699)
$CC_{1/2}$ [a]	100.0 (75.8)
Redundancy [a]	6.1 (3.8)
**Refinement**	
Non-hydrogen atoms	
R_{work} [a, c]	0.138 (0.207)
R_{free} [a, d]	0.162 (0.210)
Average B-factor [Å^2]	14.6
Protein residues	94 / 13.5
Peptide residues	10 / 14.1
Water molecules	176 / 25.0
Buffer molecules	3 / 18.3
r.m.s.d. [e] bond length [Å]	0.016
bond angles [°]	1.628
Ramachandran outliers [%]	0
Ramachandran favored [%]	99.1

[a] values in parentheses refer to the highest resolution shell.

[b] $R_{meas} = \Sigma_h [n/(n-1)]^{1/2} \Sigma_i | I_h - I_{h,i} | / \Sigma_h\Sigma_i I_{h,i}$.where I_h is the mean intensity of symmetry-equivalent reflections and n is the redundancy.

[c] $R_{work} = \Sigma_h | F_o - F_c | / \Sigma F_o$ (working set, no σ cut-off applied).

[d] R_{free} is the same as R_{work}, but calculated on 5% of the data excluded from refinement.

[e] Root-mean-square deviation (r.m.s.d.) from target geometries.

Supplementary Table 2 Peptide interactions with a distance cut-off of ≤ 3.3 Å.

***dm*Spinophlin-PDZ**	***dm*Neurexin**	**distance**	**Peptide**	**Peptide**	**distance**
$Leu1271^{N}$	$Val1840^{O}$	2.7			
$Leu1273^{N}$	$Val1840^{OXT}$	3.3			
$Leu1273^{O}$	$Val1840^{N}$	2.9			
$Arg1335^{NE}$	$Thr1839^{O}$	3.0			
$Arg1335^{NH}$	$Thr1839^{O}$	2.9			
			$Tyr1839^{OH}$	$Glu1837^{OE2}$	2.6
$Ile1275^{N}$	$Trp1838^{O}$	2.9			
$Ile1275^{O}$	$Glu1837^{N}$	2.9			
$Lys1294^{NZ}$	$Glu1837^{OE1}$	3.0			
$Gln1327^{NE2}$	$Lys1836^{O}$	3.1			
			$Asp1834^{N}$	$Ser1832^{O}$	3.1
			$Asp1834^{OD1}$	$Asp1831^{O}$	2.9
			$Asp1834^{OD1}$	$Asp1831^{OD1}$	2.8
$Lys1288^{NZ}$	$Ser1832^{OG}$	3.1			
			$Asp1831^{O}$	$Asp1831^{OD1}$	2.9

Supplementary Methods

Protein expression and purification

Protein expression was performed using BL21-T1^R cells. Cells were grown in auto-induction ZY-medium (Studier, 2005) with Kanamycin for 4 h at 37 °C. Afterwards, the temperature was decreased to 18 °C and cells were grown overnight. Harvested cells were resuspended in extraction buffer (40 mM Tris/HCl (pH 7.5) 400 mM NaCl, 1 mM DTT, 10 mg/l lysozyme and 5 mg/l DNase I) at room temperature and subsequently lysed by sonification. Lysates were centrifuged at 56,000 x g for 45 min to pellet the cell debris. Supernatants were subjected to affinity chromatography using amylose resin (NEB). Two washing steps were performed using washing buffer (20 mM Tris/HCl (pH 7.5) 200 mM NaCl, 1 mM DTT) at room temperature. Amylose resin was incubated twice with washing buffer supplemented with 10 mM maltose for 15 min for protein elution. The MBP-tag of the PDZ domains was cleaved off using TEV protease (1 mg/ml). Protease was added to the eluted protein in a molar ratio of 1:30 and the reaction incubated at 4 °C overnight. TEV protease and cleaved His_6-MBP-tag was removed using Ni-NTA resin. TEV-cleaved constructs were purified using a Superdex 75 26/60 column (GE Healthcare), where fractions containing protein were pooled and concentrated using a Centricon (MWCO 3,000) (Millipore). Protein concentrations were determined by UV-absorption.

Isothermal titration calorimetry

Isothermal titration calorimetry experiments were performed at 25°C on an iTC200 microcalorimeter (Malvern Instruments Ltd.). A peptide with the sequence ^{1831}DSKDVKEWYV^{1840} was synthesized by JPT – Innovative Peptide Solutions company. Lyophilized peptide was resuspended in the same buffer as the proteins. Spn-PDZ was injected in steps of 30 µM equivalent concentration against 182 µM of peptide; MBP-Syd-1-PDZ was injected in steps of 18 or 19 µM equivalent concentration against 160 or 174 µM of peptide. In a control experiment, MBP with injected (22 µM equivalents) against 174 µM peptide. All measurements were performed with 20 injections of 2.0 µl volume at intervals of 2 min. The heat released was obtained by integrating the calorimetric output curves. Binding

parameters were calculated with Origin5 software using the “One Set of Sites” curve-fitting model provided by the software.

Thermofluor

A thermofluor analysis was performed for buffer optimization using a Mx3005P qPCR system (Agilent). Buffers containing zinc salts in different concentrations gave a thermal shift of at least 12 °C.

Crystallization and crystal cooling

For crystallization, Spn-PDZ was purified as described, with 100 µM zinc chloride present in all buffers. The protein was concentrated to 60 mg/ml. The unsolubilized peptide (DSKDVKEWYV) was mixed in a three-fold molar excess with the protein and incubated for 2 h on ice. Insoluble peptide was removed by centrifugation (16,000 g for 1 min) prior to the crystallization experiments. The initial crystals were obtained by the sitting-drop vapor-diffusion method at 18 °C with a reservoir solution composed of 0.1 M Tris-HCl (pH 8.5 at RT), 0.01 M nickel chloride and 20 % (w/v) PEG 2000 MME. Crystals were cryo-protected using 20 % (v/v) ethylene glycol, which was added to the reservoir solution.

X-ray data collection, structure determination and refinement

Synchrotron diffraction data were collected at beamline 14.2 of the Joint Berlin MX Laboratory at BESSY (Berlin, Germany). X-ray data collection was performed at 100 K. Diffraction data were processed with XDS[1]. The structure of Spn-PDZ in space group $P4_32_12$ was solved by molecular replacement using Phaser-MR[2], in which the shortened structure of the Spn PDZ domain from *Rattus norvegicus* (PDB entry 3EGG[3]) was used as a search model. A randomly generated set of 5 % of reflections was excluded from the refinement for the calculation of the free R-factor. The structure was initially refined by applying a simulated annealing protocol and, in later refinement cycles, by maximum-likelihood restrained refinement using PHENIX[4, 5]. Model building and water picking was performed with COOT[6]. Model quality was evaluated with MolProbity[4, 7]. Figures were prepared using PyMOL[8].

Detailed methods for GCamp5 imaging; assaying spontaneous and evoked release by Ca^{2+} imaging.

Third instar larvae of both sexes expressing UAS-myrGCaMP5[9] in the muscle were dissected in Ca^{2+} free, ice-cold HL3 saline containing (in mM): NaCl 70, KCl 5, $MgCl_2$ 20, NaHCO3 10, trehalose 5, sucrose 115, Hepes 5 (pH 7.2) at room temperature). The motoneuron nerves were cut below the ventral nerve cord and the CNS was removed. The preparation was allowed to rest for 5-10 min in HL3 containing 1.5 mM $CaCl_2$ at RT. During this time, the motor nerve of the respective segment was sucked into a stimulation pipette filled with HL3 for later NMJ stimulation. Image sequences (spontaneous and evoked release) were acquired at 20 Hz with an Olympus BX51WI epifluorescence microscope with a 40x (NA 0.8) water immersion objective (Olympus), equipped with a Lambda DG-4 light source (Sutter Instruments, Novato, CA, USA) and a Hamamatsu Orca-Flash 4.0 V2 camera (exposure time 0.05 s). The camera was operated in stream mode using HoKaWo software (vers. 2.9, Hamamatsu Germany). First, spontaneous activity in muscle 4 in segments A2 or A3 was recorded for 100 s. Then evoked release was stimulated 35 times by depolarizing the afferent motor nerve using voltage steps to 10 V. Each step lasted 300 µs and was applied at a frequency of 0.2 Hz with an S48 Stimulator (Grass Technologies, Warwick, RI, USA). The stimulator and camera were triggered using a Digidata 1440A (Axon CNS, Molecular Devices, Sunnyvale, CA, USA), running Clampex software (vers. 10.4, Molecular Devices, UK). The larval fillet was fixed immediately in PBS containing 4 % PFA immediately after the final stimulation was applied. Fillets were then stained for BRP(see above).

Ca^{2+} imaging and data analysis.

Image sequences were processed using ImageJ (version 1.48t and 1.48q). Slight drift between images was corrected with the “TurboReg” plugin (http://bigwww.epfl.ch/thevenaz/turboreg/), which uses the “Rigid Body” transformation[10] to register all images to the first frame of the spontaneous recording. Additionally, maximal z-projections of post-hoc confocal images of the GCaMP5 fluorescence were aligned to this target frame using the

“affine” transformation. The same transformation was also applied to the other channel with the BRP staining (Supplementary Fig.10). Single AZs were automatically identified through their intensity maxima and equally-sized regions of interest (ROIs, 0.65 µm diameter) were placed around each maximum (Supplementary Fig.10). Integrated fluorescence intensity values from each ROI were read out from each frame to capture the temporal change in GCaMP5 fluorescence at a particular AZ. Each ROIs was then moved to a region outside of the GCaMP5 signal to obtain a background intensity value, which was subtracted from the signal. Data were transferred to Matlab (Mathworks, vers. R2011a) for further analysis. Spikes were detected by analyzing the background corrected GCaMP5 intensity profiles using a custom-written script. Traces were filtered using a running average filter implemented in the Matlab function “filter” with a box size of four frames. Spikes were identified by detecting signals that exceeded the standard deviation of the signal by a factor of 4 for at least three consecutive frames. The following criteria were applied to prevent the same signal being counted several times at adjacent AZs: only the largest signal in recordings of spontaneous activity was considered if several AZs had simultaneous fluorescence peaks. Signals were only considered for the analysis of evoked episodes if they were temporally locked to the stimulation, with peak values occurring within 1 s after the stimulus. Spikes that coincided at different AZs were only considered if they were at least 2.5 µm apart, otherwise only the largest signal was considered. All signals matching these criteria were evaluated by visual inspection and peaks that showed atypical rise and decay kinetics were rejected. A section of the local GCaMP5 fluorescence signal (2 s prior and 2s after the maximal spike value) was selected to obtain the average GCaMP5 response (Fig. 7c and d) and the baseline corrected by subtracting a line that was fitted to the fluorescence signal during the first and last second of this 4 s window. Traces were then averaged over all events from all AZs in one animal and, finally, averaged over all animals. The total number of spontaneous events per NMJ was divided by the number of analyzed AZs and the acquisition time in order to calculate the frequency of spontaneous events per AZ. The total number of AZs that showed activity at least once

was divided by the total AZ number to obtain the fraction of active AZs. The release probability per AZ was calculated by dividing the total number of stimulus-locked, evoked events per NMJ by the number of AZs and stimuli. All values were then averaged over all animals. The BRP intensities per AZ were measured from confocal maximal projection images using the same ROIs that were used for reading out the GCaMP5 fluorescence. The BRP intensities were then binned in ascending order in five bins containing the same number of AZs and the average BRP intensity as well as the average number of evoked events calculated per AZ. Binned data were then averaged over all animals of one group. Linear fits were performed in OriginPro 6G (vers. 8.0773) taking vertical and horizontal error bars into account. The function “Compare Datasets” was used to test whether the two datasets were significantly different from one another.

Supplementary References

1. Kabsch W. Xds. *Acta Crystallogr D Biol Crystallogr* **66**, 125-132 (2010).

2. McCoy AJ, Grosse-Kunstleve RW, Adams PD, Winn MD, Storoni LC, Read RJ. Phaser crystallographic software. *J Appl Crystallogr* **40**, 658-674 (2007).

3. Ragusa MJ, Dancheck B, Critton DA, Nairn AC, Page R, Peti W. Spinophilin directs protein phosphatase 1 specificity by blocking substrate binding sites. *Nat Struct Mol Biol* **17**, 459-464 (2010).

4. Adams PD*, et al.* PHENIX: a comprehensive Python-based system for macromolecular structure solution. *Acta Crystallogr D Biol Crystallogr* **66**, 213-221 (2010).

5. Afonine PV*, et al.* Towards automated crystallographic structure refinement with phenix.refine. *Acta Crystallogr D Biol Crystallogr* **68**, 352-367 (2012).

6. Emsley P, Lohkamp B, Scott WG, Cowtan K. Features and development of Coot. *Acta Crystallogr D Biol Crystallogr* **66**, 486-501 (2010).

7. Laskowski RA, Macarthur MW, Moss DS, Thornton JM. Procheck - a Program to Check the Stereochemical Quality of Protein Structures. *Journal of Applied Crystallography* **26**, 283-291 (1993).

8. DeLano WL. The PyMOL Molecular Graphics System (2002).

9. Melom JE, Akbergenova Y, Gavornik JP, Littleton JT. Spontaneous and evoked release are independently regulated at individual active zones. *J Neurosci* **33**, 17253-17263 (2013).

10. Thevenaz PT, Ruttimann UE, Unser M. A Pyramid Approach to Subpixel Registration Based on Intensity. *IEEE TRANSACTIONS ON IMAGE PROCESSING* **7**, (1998).
